# Sex- and age-specific aspects of human peripheral T-cell dynamics

**DOI:** 10.3389/fimmu.2023.1224304

**Published:** 2023-10-13

**Authors:** Justyna Mika, Kengo Yoshida, Yoichiro Kusunoki, Serge M. Candéias, Joanna Polanska

**Affiliations:** ^1^ Department of Data Science and Engineering, Silesian University of Technology, Gliwice, Poland; ^2^ Department of Molecular Biosciences, Radiation Effects Research Foundation, Hiroshima, Japan; ^3^ Université Grenoble Alpes, Commissariat à l'Energie Atomique et aux Energies Alternatives (CEA), Centre National de la Recherche Scientifique (CNRS), Interdisciplinary Research Institute of Grenoble (IRIG), Laboratory of Chemistry and Biology of Metals (LCBM), Grenoble, France

**Keywords:** TCR, repertoire diversity, aging, modelling, homeostasis

## Abstract

**Background:**

The diversity of the antigenic T cell receptor (TCR) repertoire clonally expressed on T lymphocytes is a key element of the adaptive immune system protective functions. A decline in diversity in the older adults is associated with health deterioration. This diversity is generated by the rearrangement of TRB genes coding for TCR chains during lymphocyte differentiation in the thymus, but is essentially maintained by peripheral T lymphocytes proliferation for most of life. Deep sequencing of rearranged TRB genes from blood cells allows the monitoring of peripheral T cell repertoire dynamics. We analysed two aspects of rearranged TRB diversity, related to T lymphocyte proliferation and to the distribution of the T cell clone size, in a collection of repertoires obtained from 1 to 74 years-old donors.

**Results:**

Our results show that peripheral T lymphocytes expansion differs according to the recombination status of their TRB loci. Their proliferation rate changes with age, with different patterns in men and women. T cell clone size becomes more heterogeneous with time, and, in adults, is always more even in women. Importantly, a longitudinal analysis of TRB repertoires obtained at ten years intervals from individual men and women confirms the findings of this cross-sectional study.

**Conclusions:**

Peripheral T lymphocyte proliferation partially depends on their thymic developmental history. The rate of proliferation of T cells differing in their TRB rearrangement status is different in men and women before the age of 18 years old, but similar thereafter.

## Introduction

The adaptive immune system plays a central role in the defense of the organism against pathogens and cancers throughout life through the ability of peripheral T lymphocytes to recognize their cognate antigens via their clonally distributed antigenic T cell receptor (TCR). The maintenance of a very large and diverse repertoire of TCRs is paramount to maintaining efficient protection against pathogens and tumour development (1–3).

TCR repertoire diversity is created by the assembly of TRA and TRB genes coding for the TCRα and β subunits, respectively, in developing lymphocytes. They are created by site-specific genetic recombination from arrays of variable (V), diversity (D, only for TRB) and joining (J) genes borne on specific chromosomal loci. Diversity results from the large number of possible V, D and J combinations and is further increased by the molecular processing of V, D and J gene ends before their ligation (1–3). Because of the essentially random nature of the end processing, only one out of three rearrangements will be productive, i.e. maintains an open reading frame between the V and J genes and gives rise to a protein.

Productive TRB gene recombination is required for immature lymphocytes maturation in the thymus (4). When rearrangement of TRBV, D and J genes on the first allele is non-productive, the cells will rearrange TRB genes on their second chromosome to pass this so-called β-selection checkpoint and resume their differentiation (4). If this second attempt is still non-productive, these cells will die from the absence of TCR-mediated survival signals. Thus, peripheral T cells may carry one productively rearranged TRB locus while the other in germ-line configuration [TRB(+/GL) cells], or two rearranged TRB loci, one non-productively and one productively [TRB(-/+) cells]. We recently showed in mice that TRB(+/GL) peripheral T lymphocytes have a competitive advantage over their TRB(-/+) counterparts, and their proportion increases with age (5).

The human peripheral T lymphocyte population represents around 10^12^ cells. In young adults, the naïve T lymphocytes population carries a minimum of 0.6 to 1.2 x 10^8^ different αβTCRs (6). In mice, the maintenance of the naïve peripheral T cell pool heavily relies on *de novo* production of T lymphocytes by the thymus. However, in humans, homeostatic proliferation is by far the main driver of this maintenance, even at a relatively young age (7–10). Homeostatic proliferation contributes for two thirds to the maintenance of peripheral T cells in the two first decades of age (7), and for more than 95% for naïve T cells in older adults (11, 12). Age-and sex-dependent thymic atrophy concomitantly reduces thymic contribution to the naïve T cell pool in adults (13). Peripheral T lymphocytes differentiate into memory cells after antigen-specific, TCR-driven immune responses. The diversity of the TCR repertoire in memory T lymphocytes is around 100 times smaller than in naïve T lymphocytes (14). The mean T lymphocyte clone size, estimated by rearranged TRB gene sequencing, is much larger in memory than in naïve CD4^+^ and CD8^+^ T cells and is generally larger for CD8^+^ than CD4+ memory cells (6). However, the richness of the rearranged TRB gene repertoire is on average 5 times higher in peripheral CD4^+^ than CD8^+^ T lymphocytes, irrespective of the donor’s age (15).

The repertoires of rearranged TR genes generated in CD4^+^ and CD8^+^ thymocytes are different (16). As the respective abundance of naïve and memory CD4^+^ and CD8^+^ T lymphocyte populations evolves in time (12, 17, 18), the composition and diversity of the peripheral TCR repertoire further changes with age (6, 12, 19, 20). In addition, differences in CD4^+^ and CD8^+^ T lymphocytes output and in the numbers, proliferation and activation of peripheral blood CD4^+^ and CD8^+^ T lymphocytes (13, 20–23) also induce differences in the repertoire of rearranged TRB genes in men and women (20, 24, 25).

These changes in TCR repertoire may have important physiological consequences. A reduced diversity of peripheral T cell TCR repertoire, resulting in a reduced ability to recognize antigens (10), probably contributes to the decline of immune system’s fitness with age, thought to be responsible for the increased frequency and severity of infectious diseases and cancer in older adults (26–28). At the same time, homeostatic proliferation of T cell clones with potentially promiscuous TCR specificities may lead to the development of autoimmune diseases, more frequent in women and older adults (22, 29, 30).

In this study, to gain insights into the age- and sex-specific dynamics of the T cell repertoire, we modelled changes in the frequency of productively rearranged TRB genes, reflecting T lymphocyte proliferation, and in the distribution of unique rearranged TRB genes, reflecting T lymphocyte clonality, in a cohort of 487 men and women from infancy to old age (31) and in a smaller group of individual adult men and women who gave blood three times at 10 years intervals (25). Our results show that the proliferation of peripheral blood T lymphocytes is affected by their differentiation history, and the diversity of the peripheral T cell population is differentially maintained in different periods of life in men and women.

## Materials and methods

### Generation of data

All the rearranged T cell receptor β chain gene (TRB) repertoires analysed in this study have been generated by Adaptive Biotechnologies (Seattle, WA, USA) by high throughput sequencing following multiplex amplification of all possible V(D)J combinations. After sequencing, immunoSEQ Analyzer software (Adaptive Biotechnologies) was used to automatically annotate rearranged TRB genes and their complementary determining regions 3 (CDR3). A cross-sectional dataset of rearranged TRB gene repertoires generated from 587 donors aged from 1 to 74 years was downloaded in June 2017 from the Adaptive Biotechnologies website^1^ (31). A second longitudinal dataset was obtained from 6 donors with informed consent, sampled 3 times each, around 10 years apart as described (25). The characteristics of both sets are provided in **Table 1**.

**Table 1:** Characteristics of the datasets.

	Cross-sectional dataset (31)	Longitudinal dataset (25)
Number of samples	587	36
Age range	from 1 to 74 years	from 24 to 65 years
Sex information	Women: 256/44% Men: 308/53%	Women: 3/50% Men: 3/50%
Time-course	–	3 timepoints ~ 10 years apart
Type of repertoire	whole blood	sorted blood CD4^+^and CD8^+^ T cells

### Data curation

Samples with no information about age or sex of the donors were excluded from further analysis (96 samples in the cross-sectional dataset). Then based on the total number of sequences, outlying samples were detected according to Bruffaerts criterion for highly skewed distributions (32) and filtered out. As a result, 487 and 36 samples were left in the cross-sectional and longitudinal sets for analysis, respectively (**Table 2**). The resulting curated cross-sectional dataset is composed of 228 TRB repertoires from women and 259 TRB repertoires from men.

**Table 2:** Summary of data cleaning and pre-processing.

		Cross-sectional dataset (31)		Longitudinal dataset (25)	
Initial number of sequences (*seq*)/repertoires (*rep*)		3 076 345 432 *seq* /587 *rep*		70 960 689 *seq* /36 *rep*	
Repertoires filtration	No information about age or sex	349 401 766 *seq*/96 *rep*	11.36%	0 *seq* /0 *rep*	–
	Outliers	95 231 561 *seq* /4 *rep*	3.10%	0 *seq* /0 *rep*	–
Rearranged TR sequence filtration	V orphon genes	190 889	0.01%	5 625	0.01%
	Unidentified V genes	682 209 485	22.18%	13 446 297	18.95%
	Unidentified J genes	1 125 798	0.04%	23 268	0.03%
	Indexing errors^#^	5 166 024	0.17%	93 416	0.13%
	Dataset curation^$^	97 667 915	3.17%	2 868 170	4.04%
**Final number of sequences (*seq*)/repertoires (*rep*)**		**1 845 351 994 *seq*** **/487 *rep***		**54 523 913 *seq*** **/36 *rep***	

Total number of sequences are reported (seq). Percentages refer to initial number of sequences for corresponding sets. Whole repertoires were filtered only within the Repertoires filtration step of pre-processing.

^#^Indexing errors refer to sequences in which the annotated nontemplated N2 region of CDR3 starts at a position preceding the start of CDR3 region.

^$^Dataset curation refers to filtration of sequences involving V or J genes classified by IMGT as non-functional or open reading frame.

The IMGT^®^ reference database (as accessed 06/09/2019)^2^ was used for identification of functional TRBV, TRBD and TRBJ gene in rearranged TRB genes. Sequences using orphon V genes, sequences with un-identified V or J gene, sequences with indexing errors and sequences involving V or J genes classified as non-functional or open reading frame were filtered out from each TRB repertoire (**Table 2**). Finally, the frequency of each V and J gene was checked in all samples of this curated dataset. In a few repertoires, single V genes were not found, and TRBV07.3 was not used in 5 donor from the cross-sectional cohort. TRBV03-01 was extremely rare (mean frequency 0.002%, range 0.00003% to 0.089%) and was not found in 91 and 13 samples of the cross-sectional and longitudinal datasets respectively. Rearranged TRB sequences using TRBV03-01 were filtered from further analysis. Altogether, data curation resulted in a collection of repertoires of rearranged TRB sequences composed of a common set of 42 V genes, 13 J genes, and 2 D genes. For each repertoire, sequences were grouped into productive and non-productive subsets, based on open CDR3 reading frame.

A clone is defined a group of rearranged TRB genes with the same nucleotide sequence or, by extension, a group of T lymphocytes sharing the same TCRβ chain.

### Pre-processing

The longitudinal set consisted of rearranged TRB gene repertoires obtained from CD4^+^ and CD8^+^ peripheral T lymphocytes sorted from PBLs cryopreserved at the time of sampling from 6 donors (25). In order to compare the effects of age and/or sex in these donors with data from the cross-sectional set, we merged samples from longitudinal set for each donor at each time point, based on the CD4/CD8 ratio determined by FACS at the time of cell sorting (**Supplementary Information 1**). A downsampling pipeline was developed for this merging process, in which the drawing of the expected number of sequences from the overrepresented CD4 or CD8 sample was performed. This number is calculated as a function of the CD4/CD8 blood ratio for each sample and the total number of rearranged TRB gene sequences generated from the sorted CD4^+^ and CD8^+^ T lymphocyte populations. Next, the sequences are drawn from the subset of cells for which the expected number of sequences was smaller than actually observed (see **Supplemental Information 1** for details). Thus, a whole blood TCR repertoire sample is reconstituted by merging the selected numbers of CD4 and CD8 sequences and is used for further data curation and pre-processing, as described above, and analysis. The whole process is repeated 100 times to diminish the impact of random sampling on the results. This downsampling pipeline was applied for longitudinal set only.

### Diversity estimation

Pielou’s J index was chosen as a measure of diversity to compare several parameters of the rearranged TRB repertoire (5, 33) as we found it to provide stable results for a wide range of sequencing coverage (see paragraph *Determining the impact of sequencing depth below* and **Supplementary Materials**). It is calculated as follow (Equation 1):


(Equation 1a) $$\text{J = }\frac{\text{H}}{\text{lnS}}$$

where S denotes number of classes and H - Basharin’s unbiased estimator of Shannon’s entropy calculated as:


(Equation 1b) $$\text{H}=-\sum_{i=1}^{S}p_{i}{\text{lnp}}_{\text{i}}-\frac{S-1}{2\text{N}}$$

where ${\text{p}}_{\text{i}}$ denotes frequency of i-th class and N – total number of sequences in a group.

Pielou’s J index takes values from 0 (one class dominating) to 1 (all classes evenly distributed). It estimates the evenness of the distribution and allows for comparison of groups of different number of classes, i.e. repertoires with different number of TRB sequences.

Diversity of functionality status of rearranged TRB gene sequences, further denoted as Status Diversity, was calculated as Pielou’s J index considering for every sample two classes of events: productive and non-productive TRB rearrangements (S = 2, p_1_ = frequency of productive sequences and p_2_ = frequency of non-productive sequences, N – total number of productive and non-productive sequences in a sample; Equation 1b). Every copy of a unique rearrangement was considered to calculate the total number of productive and non-productive rearrangements. Diversity of unique TRB sequences, further denoted as Sequence Diversity, was calculated as Pielou’s J index considering the number of copies of every unique sequence in a sample (S = number of unique TRB clones, p_i_ – frequency of a unique clone, N – total number of all clones sequenced in a sample). In the case of the longitudinal dataset, the average value of diversity indices from 100 repeats of the downsampling procedure served as a final estimate of Status Diversity and Sequence Diversity.

### Response modelling

For the cross-sectional study, weighted linear regression was applied to model relations of Status Diversity and Sequence Diversity with age ( $Diversity=\beta_{0}+\beta_{1}AGE$ ) in donors (Equation 2 for Status Diversity and Equation 3 for Sequence Diversity in the Results section). Total counts of TRB sequences for every sample served as weights, valuing more samples with higher sequencing coverage. Next, a piecewise linear regression pipeline was applied to find different patterns of response in different age ranges. To find the best age cut-off value to divide donors into two subgroups, a brute-force technique was used, in which all possible splits were tested. The minimal number of donors in a subgroup was set to 5, as a minimal value to provide grounds to create a valid regression model. For every age split, a backward model selection based on Bayesian Information Criterion (BIC, calculated with BIC function in stats package in R) was used to select the optimal model. The starting model included age and sex as the explanatory variables and their first-order interaction term. For the sex variable, men served as a reference. The best age split was determined based on a minimal sum of two BIC values from the subgroup models. Weighted linear regression and piecewise linear regression pipelines were applied for all donors, as well as for men and women separately.

The comparison of the obtained age and sex subgroup-specific models included verification of the hypotheses on equality of model coefficients. A T-test was applied to compare the slope coefficients of the different models.

For the longitudinal study, linear mixed-effects modelling (34) was applied to determine the relations of Status Diversity and Sequence Diversity with age in reconstituted TRB repertoire. We assumed that random variation in models is provided by separate donors, whereas fixed-effects may come from the impact of age or sex. For both diversity measurements, backward model selection based on likelihood ratio test value was utilized. The starting model included age and sex as the explanatory variables and their first-order interaction term with additional consideration of random-effects provided by donors. For the sex variable, men served as a reference. For every model, the intraclass correlation coefficient (ICC) was calculated to estimate what part of variance comes from individual changes.

In the case of separate models for CD4^+^ and CD8^+^ cell subsets, the linear mixed modelling pipeline described above was applied with additional parameters being checked. Here we assumed, that random variation in models is provided by separate donors and cell type, whereas fixed-effects may come from the impact of age, sex, cell type, and/or interactions of them. For cell type variable, CD4^+^ cells served as a reference. For every model, intraclass correlation coefficients were calculated, estimating what part of variance comes from individual changes and cell type changes.

### Determining the impact of sequencing depth

The distribution of sequencing coverage among donors from cross-sectional set was quite diverse (**Supplementary Figure 1**, **Supplementary Table 1**). The total counts of sequences after data curation ranged from 526,247 to 8,109,134. It was observed that lower sequencing coverage results in higher Pielou’s value (calculated as the normalized Shannon’s entropy, without the Basharin’s adjustment). Thus, first to diminish the impact of small coverage, the Basharin’s unbiased estimator of entropy was calculated (Equation 1b). Moreover, separate models were created after data subsampling to obtain the same determined sequencing depth for all repertoires. Following values of coverage (i.e. total counts of sequences after the data curation step) were chosen: 10,000, 80,000, 150,000, 500,000, 1,000,000, 2,000,000 and 4,000,000. The rearrangements were subsampled from the known distribution of sequences for a given sample until the determined total counts of rearrangements were reached. All samples had at least 500,000 sequences. However, for the thresholds of 1, 2 and 4 million of total sequences, only a fraction of samples had at least that many sequences measured (469 samples had at least 1 million of total counts; 378 samples had at least 2 million of total counts and 219 samples had at least 4 million of total counts). In these instances, the subsampling was performed only for the samples that had at least the determined threshold of total counts of rearrangements. The procedure of subsampling was performed for every sample and every sequencing coverage 100 times. Next, Status Diversity and Sequence Diversity were measured for every repeat of subsampling as explained in the *Diversity estimation* section above. The models in time were created considering average values of Status Diversity and Sequence Diversity for a given coverage.

## Results

### Status Diversity in age

**Figure 1:**
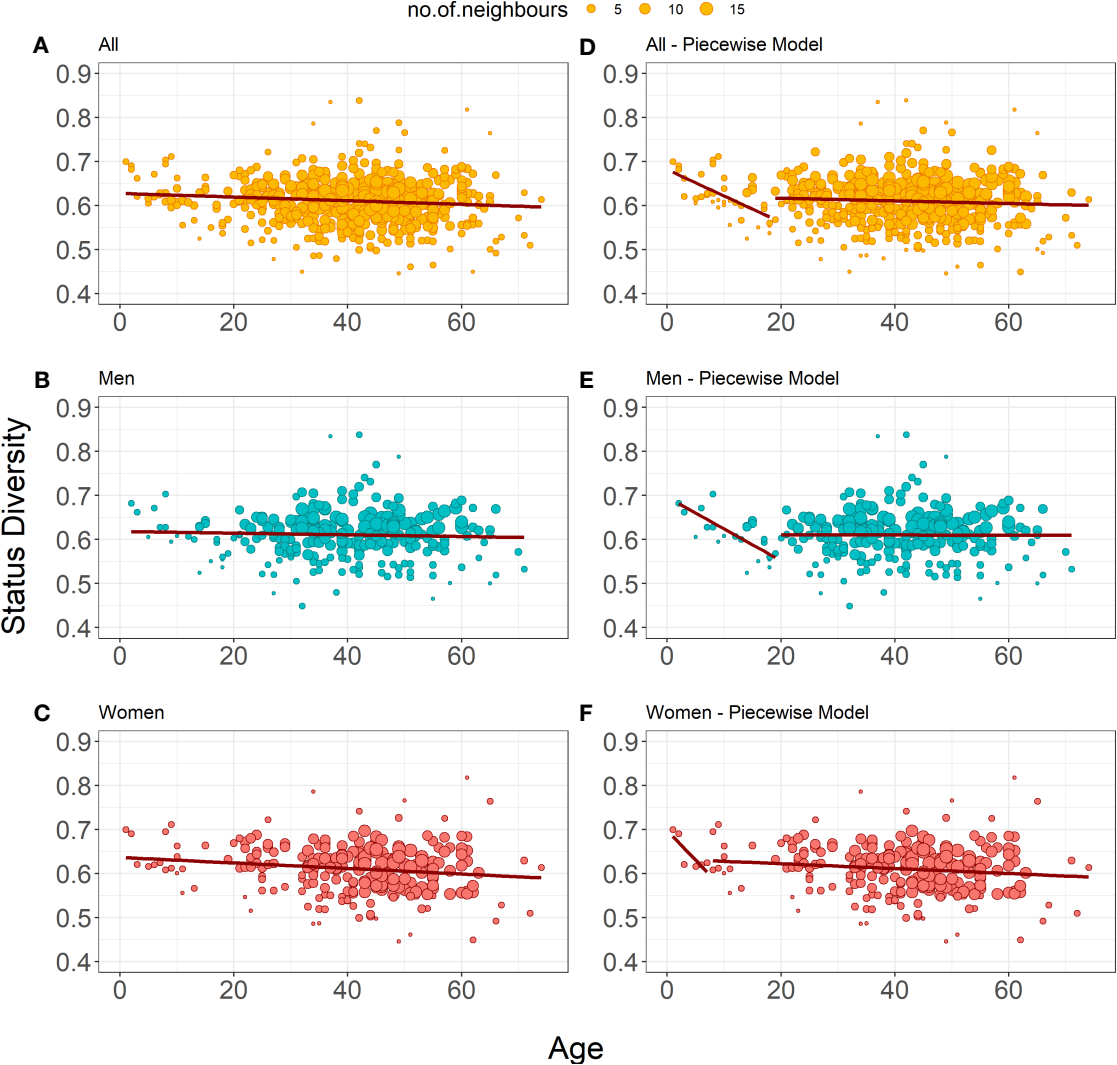
Bubble plots representation of Status Diversity weighted linear regression models. **(A-C)** Show the weighted linear regression model for all donors (n=487), only men (n=259) and only women (n=228), respectively. **(E, F)** Show piecewise weighted linear regression for all donors, only men, and only women respectively. The red line on all plots shows the evolution of Status Diversity as calculated by Equation 2. The size of a dot depends on the local density of similar results (the bigger the dot, the more neighboring samples with similar values for diversity and age).

Changes in the ratio of productive to non-productive rearranged TRB genes in peripheral blood T cells indicate a differential abundance of TRB(+/GL) and TRB(-/+) lymphocytes. We used Pielou’s diversity index (see Equation 1 in Methods section) for the productive *vs* non-productive status of the rearranged TRB sequences, referred to as Status Diversity, to analyse these changes over time in a dataset of 487 unrelated donors. A lower Status Diversity value denotes a more heterogeneous distribution of sequences subsets, which can result only from an increased frequency of productive rearranged TRB genes, resulting from either proliferation of TRB(+/GL) cells or loss of TRB(-/+) cells. Weighted linear regression analysis:


(Equation 2) $$Status\;Diversity=\;\beta_{0}+\beta_{1}*AGE$$

showed a slight but significant decrease in Status Diversity with age in this dataset (**Table 3**, **Figure 1A**). Separate weighted linear regression models for male and female donors were constructed for men and women. Notably, only the model for women showed a significant decrease in Status Diversity with age (p = 0.014, **Figure 1B**) while we do not observe any significant impact of age in men (**Figure 1C**). In other words, the proportion of productive sequences increases with age in women, while it is stable over time in men. Thus, peripheral TRB(+/GL) T lymphocytes preferentially expand with time over TRB(-/+) cells in women but not men or, alternatively, that TRB(-/+) cells are lost over time in women.

**Table 3:** Final weighted linear models for Status Diversity for cross-sectional dataset.

Cross-sectional dataset	N	b_0_	b_1_	Model p-val	r
All donors	487	0.6276	-0.0004	0.0221	-0.097
**All donors – (1-18 years)**	39	0.6817	-0.0060	<0.0001	-0.604
**All donors – (19-74 years)**	448	0.6227	-0.0003	0.1985	-0.062
Women	228	0.6367	-0.0006	0.0140	-0.156
**Women – (1-7 years)**	6	0.6976	-0.0134	0.0546	-0.805
**Women – (8-74 years)**	222	0.6335	-0.0006	0.0515	-0.125
Men	259	0.6183	-0.0002	0.4540	-0.040
**Men – (2-19 years)**	23	0.6962	-0.0072	<0.0001	-0.704
**Men – (20-71 years)**	236	0.6110	-0.00002	0.9459	-0.033

Both general and piecewise regression models are shown. N corresponds to number of donors. Model p-value was obtained from F test, Correlation r was calculated between AGE and Diversity variables.

We applied a piecewise regression pipeline on the Status Diversity of the TRB repertoire of all donors, and then separately for women and men repertoires only, to determine whether this effect is regular in time span, or whether it is different in youth and older age. Considering the whole population, the best age split was between people of 18 and 19 years of age. Younger donors have a significant drop in Status Diversity (r = -0.604), which stays stable in older donors (r = 0.062, **Table 1**, **Figure 1D**). Analysis of separate models for men and women showed that this general split is driven by men, as we observe a significant decline in Status Diversity with age only in men below 19 years of age. (**Figure 1E**). This result indicates that the homeostatic proliferation of T lymphocytes is not homogeneous throughout life in men. For women, piecewise regression forced the split between the age of 7 and 8 years, but the young donors’ group included only 6 individuals. Furthermore, none of the models generated by this split are statistically significant (p = 0.055 and p = 0.052, **Table 1**, **Figure 1F**). Thus, in contrast to men, there is a slight but even decrease in Status Diversity in women throughout the whole age range (**Figure 1C**), indicating that the proportion of TR(+/GL) peripheral T lymphocytes increases throughout life in women.

### Sequence Diversity in age - 526

**Figure 2:**
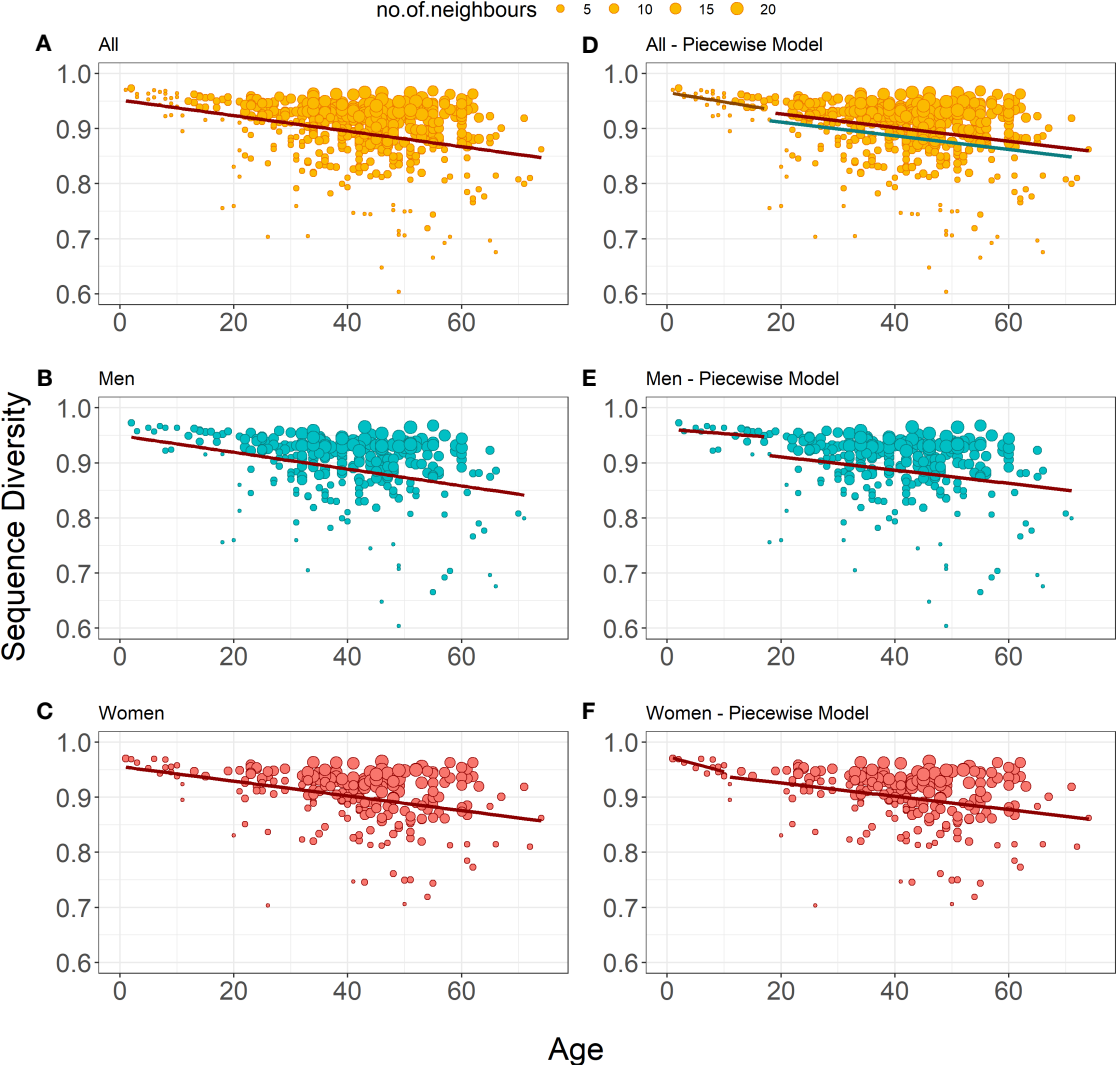
Bubble plots representation of Sequence Diversity weighted linear regression models **(A-C)** show the weighted linear regression model for all donors (n=487), only men (n=259) and only women (n=228), respectively. **(E-F)** show piecewise weighted linear regression for all donors, only men, and only women respectively. The red line on all plots shows the evolution of Status Diversity as calculated by Equation 3. On panel d, the grey line shows model for young male and female donors, the red line shows estimated model for adult women, whereas blue line depicts model for adult men, as calculated by Equation 4. The size of a dot depends on the local density of similar results (the bigger the dot, the more neighboring samples with similar values for diversity and age).

Next, we focused on the dynamics of Sequence Diversity, which reflects the relative abundance of the different TRB clones, normalized by the number of all clones in a sample. This parameter is therefore related to the clonality of rearranged TRB genes. A high Sequence Diversity value denotes an even distribution of unique TRB sequences, and a lower value indicates a more unequal distribution, with clones becoming over- or under-represented. This analysis was restricted to productive TRB sequences to account for TR-specific driven changes. A weighted linear regression analysis:


(Equation 3) $$Sequence\;Diversity=\;\beta_{0}+\beta_{1}*AGE$$

for the cross-sectional dataset showed a significant decrease of Sequence Diversity in age for all donors (r = -0.332, **Figure 2A**), as well as for men (r = -0.342, **Figure 2B**) and women (r = -0.336, **Figure 2C**) separately (**Table 4**). The models’ coefficients for men and women are not significantly different (p = 0.683 for intercept β _0_ and p = 0.590 for β _1_). Thus, the distribution of unique TRB sequences becomes more heterogeneous with age, probably because of antigen-driven T lymphocyte population changes, at the same rate in men and in women.

**Table 4:** Final weighted linear models for Sequence Diversity for cross-sectional dataset.

Cross-sectional dataset	N	b_0_	b_1_	b_2_	Model p-val	r
All donors	487	0.9519	-0.0014	0	<0.0001	-0.332
**All donors – (1-17 years)**	36	0.9660	-0.0017	0	0.0179	-0.416
**All donors – (18-74 years)**	451	0.9366	-0.0012	0.0150	<0.0001	-0.226
Women	228	0.9559	-0.0013	0	<0.0001	-0.336
**Women – (1-10 years)**	13	0.9741	-0.0028	0	0.0065	-0.671
**Women – (11-74 years)**	215	0.9500	-0.0012	0	<0.0001	-0.240
Men	259	0.9498	-0.0015	0	<0.0001	-0.342
**Men – (2-17 years)**	19	0.9613	-0.0008	0	0.3302	-0.364
**Men – (18-71 years)**	240	0.9352	-0.0012	0	0.0006	-0.239

For SEX variable men served as a reference. Both general and piecewise regression models are shown. Model p-values was obtained from F test. Correlation r was calculated between AGE and Diversity variables. N corresponds to the number of donors.

Here again, we investigated the eventual impact of age on the rate of Sequence Diversity. Piecewise regression analysis on all donors showed that the best age split was at 17 years of age (**Figure 2D**). We observe a significant drop of Sequence Diversity with age for both subsets, and the correlation with age is higher in younger donors (r = -0.416 for the younger and r = -0.226 for the older subsets). Interestingly for the donors older than 17, the best model includes information about sex to estimate the initial Sequence Diversity value (**Table 4**):


(Equation 4) $$Sequence\;Diversity=\beta_{0}+\beta_{1}*AGE+\beta_{2}*SEX$$

Thus, although the decrease of diversity for men and women proceeds in the same pace, T lymphocytes clone size distribution is more homogeneous in women. Separate piecewise regression models for only men showed a decrease in Sequence Diversity with age for donors older than 18 years; the decline in younger men was insignificant (**Figure 2E**). Women on the other hand have decreasing Sequence Diversity throughout their whole life, and this decrease is steeper at an early age (**Figure 2F**). However, there are no significant differences between the slope coefficients for younger and older donors, independently of the model: women and men together (p = 0.485, **Figure 2D**), women separately (p = 0.089, **Figure 2F**), and men separately (p=0.628, **Figure 2E**). Thus, the distribution of unique TRB sequences becomes more heterogeneous with age for men and women throughout their whole life, however, in men the process becomes significant only after the age of 18 (**Table 4**). Age has the same impact on the peripheral T lymphocyte repertoire in men and women, independently of the differences in clonality observed throughout life.

### Impact of sequencing coverage on Status and Sequence Diversity

In the cross-sectional set, the size of the repertoires ranges from over half million to over 8 million sequences. To determine if sequencing coverage might impact our modelling results, we performed a series of sequence subsamplings to defined sequence numbers (Materials and methods subsection *Determining the impact of sequencing depth).* Results of Status Diversity are shown in **Supplementary Figure 2** and **Table 2**, and Sequence Diversity in **Supplementary Figure 3** and **Table 3**, respectively. For repertoires of 10,000 to 1,000,000 sequences, the conclusions presented above for Status Diversity are largely confirmed. For the models established from 2 and 4 million sequences, even though the observations are similar, the observed models are not significant anymore. However, it must be pointed that these models were created from much lower number of samples, representing only 78% and 45% of the initial donor set, respectively, and therefore the number of datasets and the age range and distribution of the donors are different.

For Sequence Diversity our modelling results are also largely confirmed with lower values of the sequencing coverage, even if, on average, Sequence Diversity is slightly higher. The piecewise regression models resulted in a different age split for women and all donors only for coverages of 2 and 4 million sequences, but, as noted, they were obtained from lower numbers of samples with different age range and distributions.

Thus, for a defined number of repertoires, Pielou’s index provides a robust estimate of Status and Sequence Diversity evolution with age independently of sequencing coverage.

### Validation in individual donors

We next investigated changes in TRB repertoire diversity with age in a set of samples obtained from 3 men and 3 women who gave blood 3 times at about 10 years intervals, with an age range well within the boundaries of the age range of samples in the cross-sectional dataset. However, the TRB repertoire of these donors has been established separately for purified CD4^+^ and CD8^+^ T lymphocytes. Thus, we first “reconstituted” a whole blood TRB repertoire for each sample at each time point, as described in Materials and Methods. In addition, due to the repeated measures with time for these six donors, we estimated not only the variation coming from age and sex but also measured the eventual influence of interpersonal differences (random effects).

For Status Diversity, the model optimisation procedure resulted in a final model with a fixed impact of age and sex, independent of the donor. Only the intercept value covering after the individual background Status Diversity can change across the donors. The model includes first-order interaction between age and sex as described below:


(Equation 5) $$Status\;Diversity_{i}=\beta_{0}+\beta_{1}*AGE+\beta_{2}*SEX+\beta_{3}*AGE*SEX+\beta_{4,i}*DONOR_{i}$$

where *i* denotes the ID of individual donors (*i* = [1,2,3] for men and *i* = [4,5,6] for women.).

**Figure 3:**
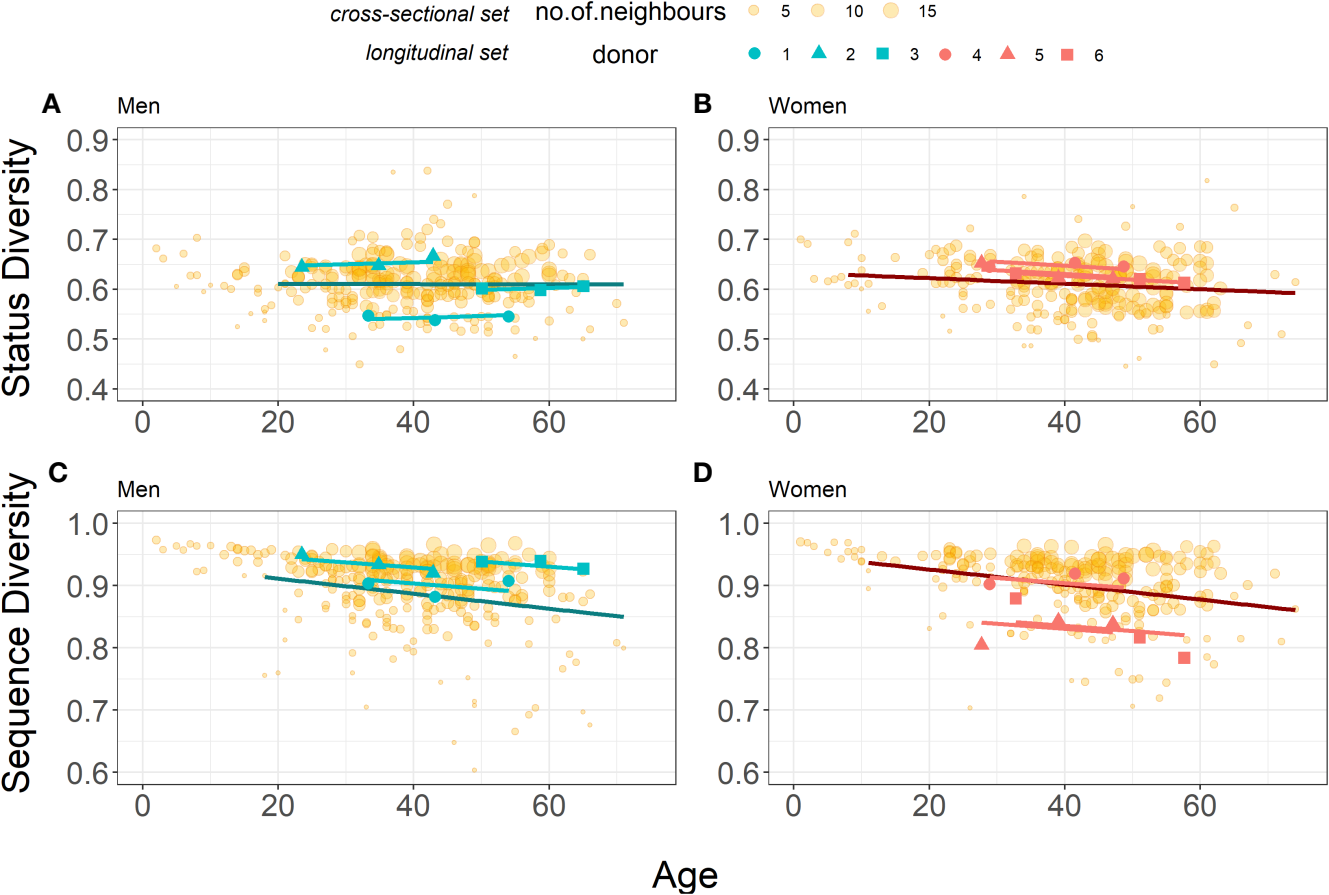
Comparison of Status Diversity **(A, B)** and Sequence Diversity **(C, D)** for the Cross-sectional (yellow points, n=259 for men and n=228 for women) and the Longitudinal (blue and pink points for men (n=3) and women n=3), respectively) datasets. Darker blue and red lines represent linear models from piecewise regression of older subset for cross-sectional set, for men and women respectively. Lighter blue and red lines represent mixed-effects models for longitudinal set (Equation 5 and Equation 6). Data for the cross-sectional set cohort are from **Figures 1**, **2**, shown again here for comparison.

The model parameters are shown in **Table 5**. A slight increase in Status Diversity with age (0.0004 per year) is observed in men. In contrast, the Status Diversity in general decreases with age (0.0007 per year) in women. The mean initial Status Diversity is higher among the women (0.6652 ± 0.0108 *vs* 0.5823 ± 0.0558 for men). The initial Status Diversity varies more across men than women (standard deviation 0.0558 *vs* 0.0108), suggesting that inter-individual differences in the frequency of productive TRB rearrangements in peripheral blood lymphocytes are bigger in men (**Figures 3A, B**). For both men and women, the major amount of variation may be explained by interpersonal differences (ICC = 95.9%). These findings are consistent with the results of the cross-sectional study (**Figures 3A, B**). The slopes of Status Diversity models for both men and women in this longitudinal analysis are similar to those calculated in our cross-sectional study (**Figures 1B, C**). These results confirm at the individual level our earlier observation of a different evolution of T lymphocyte homeostasis in men and women in the cohort of 487 donors.

**Table 5:** Final linear mixed-effects models for Status Diversity and Sequence Diversity for individual donors.

β estimates	b_0_	b_1_	b_2_	b_3_	b_4,1_	b_4,2_	b_4,3_	b_4,4_	b_4,5_	b_4,6_
**Status Diversity**	0.5823	0.0004	0.0829	-0.0011	-0.0547	0.0569	-0.0022	0.0123	-0.0044	-0.0080
**Sequence Diversity**	0.9597	-0.0008	-0.0698	0	-0.0231	0.0025	0.0206	0.0474	-0.0260	-0.0214

For SEX variable men served as a reference. *i* denotes the ID number of individual donor and *i* = [1,2,3] for men and *i* = [4,5,6] for women.

For Sequence Diversity, on the other hand, the final model consisted of a fixed impact of age and sex, independently of the donor. Donor identity only impacts the intercept value, i.e. the initial level of diversity (**Table 5**) and the model for donor *i* looks as follows:


(Equation 6) $$Sequence\;Diversity_{i}=\beta_{0}+\beta_{1}*AGE+\beta_{2}*SEX+\beta_{4,i}*DONOR_{i}$$

where *i* denotes the ID of individual donors (*i* = [1,2,3] for men and *i* = [4,5,6] for women).

The decrease of Sequence Diversity with age (0.0008 per year) is similar in men and women, but men have on average higher level of diversity (0.9597 ± 0.0220 for men *vs* 0.8899 ± 0.0411 for women). The major amount of variation may again be explained by interpersonal differences (ICC = 71.1%), but their contribution is smaller when compared to Status Diversity. The slope of this Sequence Diversity model is well within 95% confidence intervals for the age coefficient in models for cross-sectional set ((-0.0018, -0.0007) and (-0.0019, -0.0005) for men and women respectively). These findings therefore confirm in individual men and women, the results obtained for both sexes in the cross-sectional study (**Figures 3C, D**).

### Separate analysis of CD4^+^ and CD8^+^ cells

Finally, we also analysed whether age impacts similarly rearranged TRB genes Status and Sequence Diversity in CD4^+^ and CD8^+^ T lymphocytes. We applied linear mixed models on these datasets with the assumption that random variability is caused by individuals and that the effect of cell type may vary between donors. The final model for Status Diversity includes a fixed impact of age, sex, and first-order interaction between age and sex independently of the donor. The initial level of diversity is specific for donor *i* and a cell type:


(Equation 7) $$Status\;Diversity_{i}=\beta_{0}+\beta_{1}*AGE+\beta_{2}*SEX+\beta_{3}*AGE*SEX+\;\beta_{5,i}*DONOR_{i}+\beta_{6,i}*CELLTYPE$$

where *i* denotes the ID of individual donors (*i* = [1,2,3] for men and *i* = [4,5,6] for women). SEX and CELLTYPE are binary variables with men and CD4^+^ cells as reference respectively.

**Figure 4:**
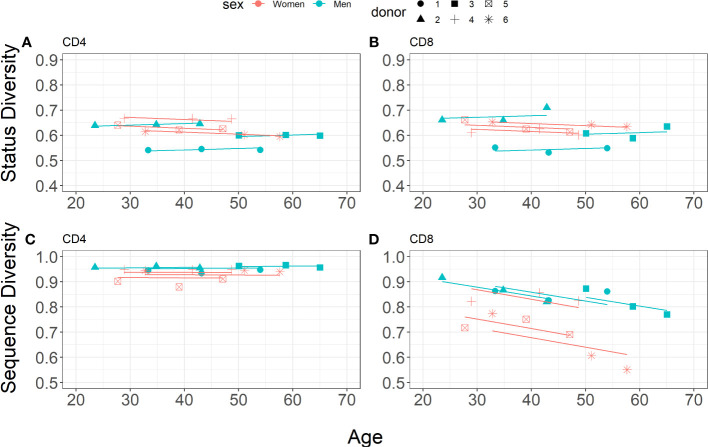
Linear mixed-effects models for Status Diversity (**A, B**, Equation 7) and Sequence Diversity (**C, D**, Equation 8) for CD4^+^and CD8^+^ cell subsets separately (blue and pink points for men (n=3) and women n=3, respectively).

The estimated parameters for CD4^+^and CD8^+^ T lymphocytes from the different donors are shown in **Table 6**. For both CD4^+^and CD8^+^ cells, Status Diversity is decreasing with age in women whereas on the opposite it slightly increases in men (0.0008 decrease per year for women *vs* 0.0006 increase per year for men). Overall, these results are in line with those found for the reconstituted repertoires (**Figure 3**). Additionally, the mean initial Status Diversity is higher among the women in CD4^+^ cells (0.6677 ± 0.0255 *vs* 0.5692 ± 0.0525 for men) and CD8^+^ cells (0.6647 ± 0.0159 *vs* 0.5826 ± 0.0683 for men, **Figures 4A, B**). A large part of the variation is explained by interpersonal differences (ICC = 63.3%) and some by cell type differences (ICC = 30.5%).

**Table 6:** Final linear mixed-effects models for Status Diversity and Sequence Diversity for CD4+ and CD8+ cells in individual donors.

Status Diversity	**b_0_**	**b_1_**	**b_2_**	**b_3_**	**b_4_**	
	0.5712	0.0006	0.0961	-0.0014	0	
	**b_5,1_**	**b_5,2_**	**b_5,3_**	**b_5,4_**	**b_5,5_**	**b_5,6_**
	-0.0528	0.0520	-0.0052	0.0286	-0.0061	-0.0212
	**b_6,1_**	**b_6,2_**	**b_6,3_**	**b_6,4_**	**b_6,5_**	**b_6,6_**
	-0.0002	0.0311	0.0094	-0.0472	0.0036	0.0344
Sequence Diversity	**b_0_**	**b_1_**	**b_2_**	**b_3_**	**b_4_**	
	0.9468	0.0002	-0.0132	-0.0003	-0.0037	
	**b_5,1_**	**b_5,2_**	**b_5,3_**	**b_5,4_**	**b_5,5_**	**b_5,6_**
	-0.0011	0.0035	0.0048	0.0083	-0.0131	-0.0009
	**b_6,1_**	**b_6,2_**	**b_6,3_**	**b_6,4_**	**b_6,5_**	**b_6,6_**
	0.0521	0.0331	0.0611	0.0402	-0.0549	-0.1032

For SEX variable men served as a reference. For CELLTYPE variable CD4^+^ cells served as a reference. *i* denotes the ID number of individual donor and *i* = [1,2,3] for men and *i* = [4,5,6] for women.

In sharp contrast, Sequence Diversity evolves differently in CD4^+^ and CD8^+^ cells over time (**Figures 4C, D**), as expected (25). The likelihood ratio test-based model optimisation procedure resulted in a final model including a first-order interaction between age and cell type, in addition to the fixed impact of age and sex, different intercept values covering after the individual and cell type driven background Status Diversity for donor *i* and first order interaction between age and sex:


(Equation 8) $$Sequence\;Diversity_{i}=\beta_{0}+\beta_{1}*AGE+\beta_{2}*SEX+\beta_{3}*AGE*SEX+\beta_{4}*AGE*CELLTYPE\;+\;\beta_{5,i}*DONOR_{i}+\beta_{6,i}*CELLTYPE_{i}$$

where *i* denotes the ID of individual donor (*i* = [1,2,3] for men and *i* = [4,5,6] for women). SEX and CELLTYPE are binary variables with men and CD4^+^ cells as reference respectively.

Thus, for CD8^+^ cells we observe a steep decrease of Sequence Diversity with time for both men and women (0.0035 per year for men *vs* 0.0038 per year for women), whereas this index is remarkably stable in CD4^+^ lymphocytes (0.0002 increase per year for men *vs* 0.0001 decrease per year for women). The mean initial Sequence Diversity is higher in men (0.9980 ± 0.0147 in CD8^+^ and 0.9492 ± 0.0032 in CD4^+^) than in women (0.9848 ± 0.0147 in CD8^+^ and 0.9317 ± 0.0107 in CD4^+^). The major amount of variation is explained by cell lineage differences (ICC = 80.0%), with a small contribution of interpersonal differences (ICC = 2.9%). Thus, for each of the donors, the distribution of unique productively rearranged TRB sequences becomes much more heterogeneous in CD8^+^ lymphocytes in 20 years, but stays unchanged in CD4^+^ T lymphocytes.

## Discussion

Due to its inherent randomness, V(D)J recombination generates both productive and non-productive rearranged TRB genes in developing thymocytes. We previously showed that murine TRB(+/GL) peripheral T lymphocytes are progressively enriched versus their (TRB(-/+) counterparts, indicating that they have a proliferation and/or survival advantage (5). This study now extends these findings to human as we observe at the population level a decrease of Status Diversity as the age of the donors increases (**Figure 1A**). This competitive advantage could result from a faster differentiation of developing T lymphocytes (5, 16). It may also result from increased TCR expression on TRB(+/GL) cells. V(D)J recombination is regulated, among others, by chromatin accessibility of the rearranging loci and genes (35). Hence, binding of transcription regulation factors (TFs) to the TRB locus may differ between rearranged (either P or NP) and germ line alleles. For example, the binding efficiency of a TF could be higher in TRB+ than GL alleles but similar between TRB- and TRB+ alleles so that the TRB+ allele in TRB(+/GL) cells may have more chance to acquire the TF than that in TRB(-/+) cells. As a consequence, the TCRB expression level may be higher in TRB(+/GL) cells than in TRB(-/+) cells, allowing a higher/faster homeostatic or activation-induced proliferation, or increased survival in the former cells. Interestingly, in old mice, CD8^+^ T cell clones with high-avidity TCRs exhibit a higher level of homeostatic proliferation than, cells with low-avidity TCRs, which are progressively lost (36). The structural avidity of TCRs for their peptide/MHC targets is in part dictated by the number of TCRs molecules engaged with peptide/MHC complexes (3). The analysis of the status of the TRB locus in the most expanded peripheral CD8^+^ T cells in relation with their TCR level could be a way to test our hypothesis that T cells which succeeded at generating a functional TRB gene at their first attempt have a competitive advantage because of their TCR expression level/avidity.

The decrease of the Status Diversity index is however uneven in age: a first period of sharp decline until the age of 18 years old is followed by a period of stability until old age (**Figure 1D**). The relative proportions of TRB(+/GL) and TRB(-/+) peripheral T lymphocytes does not evolve any longer in adulthood, either because they stop dividing or because they divide at the same rate. The dynamics of Status Diversity indeed only describes the relative abundance of TRB(+/GL) and TRB(-/+) peripheral T lymphocytes, without any consideration on their absolute rate of proliferation. The pattern observed at the population level is however the resultant of two very different situations according to the sex of the donors. Whereas age-dependent changes in Status Diversity are identical for men and the whole population of donors, no significant breakpoint could be identified for women, where Status Diversity shows a regular slow declining trend throughout life.

It is of course tempting to speculate that the sex-specific evolution of Status Diversity with age is related to sexual hormones, which influence several aspects of the development and activity of the immune system (22, 30). As the shift in Status Diversity value occurs right after puberty only in men, it could be that testosterone, directly or indirectly, suppresses the preferential expansion of naïve TRB(+/GL) cells or reduces it to the level of TRB(-/+) peripheral T lymphocytes. Androgens are indeed known to have immunosuppressive effects (37). Alternatively, it could be that it is only when testosterone levels peak that TRB(-/+) T lymphocytes catch up and expand at the same rate. In both scenarios however, the nature of the factor(s) inducing the preferential expansion of TRB(+/GL) T lymphocytes in men before the age of 20 remains to be identified.

The influence of sex hormones on this behaviour could be tested. In clinical settings as anti-hormonal therapy have been shown to restore “younger” aspects of T cell differentiation in 60 to 77 years-old men treated for prostate cancer, including a higher (38) production of new naïve T cells by the thymus. It would be interesting to investigate the evolution of Status Diversity in such patients to find out whether lowering circulating sex hormones levels impacts the relative expansion of newly generated naïve CD4 and CD8 peripheral TRB(+/GL) and TRB(-/+) lymphocytes.

Proliferation appears to be the main driver of the peripheral naïve CD4^+^ T cell development during the first 20 years of life, and their division rate slows down with age and/or the number of peripheral T cells (8, 39). The preferential proliferation of naïve TRB(+/GL) T lymphocytes might be a major contributor to the expansion and/or maintenance of the peripheral T cell pool during this period, maybe under the influence of IL-7 on CD31^+^ recent thymic emigrants (40), before reaching an equilibrium in adults where TRB(+/GL) and TRB(-/+) cells divide at the same rate. Interestingly, recent thymic emigrants have been found to be more abundant in adult women than adult men (23). Thus, the contribution of IL-7 dependent proliferation of these recent thymic emigrant could be more important on the decline of Status diversity in women and explain the difference in Status diversity evolution observed in adult men and women. These findings from our cross-sectional study for adults are largely confirmed in individual donors (**Figure 3**) over a period of 20 years of adult life, in “reconstituted” whole blood TCR repertoires and in CD4^+^ and CD8^+^ peripheral T lymphocytes analysed separately. Thus, if the signals governing the relative expansion of TRB(+/GL) and TRB(-/+) T lymphocytes involve TCR engagement with their cognate HLA molecules, TCR/CD4/HLA II and TCR/CD8/HLA I interactions and/or cytokine signalling, they produce the same effects in men but slightly favours the expansion of TR(GL/+) CD4^+^ and CD8^+^ peripheral T lymphocytes in women.

Altogether, at the population level, we observe a strong proliferation and/or survival advantage of TRB(+/GL) lymphocytes in infants and young male adults, while in female the proliferation of these peripheral T lymphocytes is rather even at all times. More work is clearly needed to characterize the TRB(+/GL) and TRB(-/+) peripheral T lymphocytes identified in this study and their specific features, including their level of TCR expression, in young and old men and women.

The dynamics of the peripheral T lymphocyte pool results from the combination of thymic output, cell death, homeostatic proliferation, antigen-driven clonal naïve T cell expansion and differentiation into memory cells. All these factors affect the representation and the size of the different T cell clones expressing a same TRB gene. The large inter-individual variability observed for Status and Sequence Diversity (**Figures 1**, **2**) in adults most probably results, at least in part, from each donor’s infectious history, leading to specific changes in their of immune repertoires. Memory CD4^+^ and CD8^+^ T cell clones are much larger than their naïve precursors (6). The number of rearranged TRB sequence declines with age in men and women (20), even though thymic output is higher for adult women than adult men (23). Sequence Diversity decline with age indicates that the distribution of rearranged TRB clone size becomes more heterogeneous with age in each donor (**Figures 2A–C**). Naïve, but not memory CD4^+^and CD8^+^ T cell clones were shown to expand in two small groups of young and aged donors (6).. Our study now shows that, at the population level, the distribution of T lymphocyte clone size changes progressively throughout life. However, quite surprisingly, Sequence Diversity declines sharply only in young girls but not boys under 17 (**Figures 2E, F**). This decline was expected, as in childhood, the immune system is probably often stimulated by *de novo* exposure to pathogens and memory T cell clones are generated, which are larger. In light of the preferential proliferation of TRB(+/GL) observed during the same period in men (**Figure 1E**), the influence of newly generated memory T cell clones on the distribution of clone size might be compensated for by a sustained proliferation of naïve T lymphocytes. This unequal proliferation could be the source of the high heterogeneity observed in the size of naïve T cell clones (41). Consequently, the size of the clones would remain homogenously high under the influence of these two effects, and it is only after the division rate of naïve cells slows down (7) that clone size distribution becomes more heterogeneous. Thus, here again, differential T lymphocyte proliferation in men and women before puberty could explain sex-specific aspects of age-related changes in the rearranged TRB repertoire in youth. From the age of 18 and until old age, Sequence Diversity declines at the same constant rate in men and women, and T lymphocyte clone size distribution is always more heterogeneous in men (**Figure 2D**), as recently observed in a correlation study on a more restricted cohort of donors (24). Age- and sex-related changes are therefore different when the representation of the different clone size (this study) or the number of different TRB genes in peripheral T cells, which transiently decreases faster in middle aged men (20) are considered.

Finally, an important point of this study is that even though the effects are small, the age- and sex-dependent evolution of the peripheral TRB repertoire are confirmed in individual donors in a longitudinal study covering a significant time span. These results will of course need to be replicated with a larger cohort of donors followed for 20 or more years. Together with the observation that the most abundant TRB clones was somewhat preserved for up to 20 years intervals (20, 25), these findings illustrate the slow evolution of the peripheral T lymphocyte population in adults. Thus, ageing always leads to a more heterogeneous TRB repertoire, with less antigenic specificities (6, 12, 20) and more differences in the respective abundance of the different T lymphocyte clones (this study). Despite a high degree of initial inter-individual variability, sex-specific differences are maintained for all adult life.

At that stage, we can only speculate on the functional consequences of these age- sex-specific changes on health, especially in old age, but they could be addressed in future studies. It will be for example interesting to analyse the Status Diversity index of pathogenic T cells in auto-immune syndromes, especially in women, to find out whether the preferential proliferation of TRB(+/GL) T cell clones could lead to the amplification of autoreactive T cells. Several studies analysed the dynamics of immune cells with aging. The level of effector memory T cells was found higher in old men than women (42, 43). As memory T cell clones are larger, this difference may contribute in the lower Sequence Diversity index observed in men. Interestingly, in women a low effector memory T cell population correlates with a lower frailty index (44, 45). One might therefore expect that a more homogenous T cell clone size distribution, i.e. a higher Sequence Diversity index, to be associated with a healthier aging and a longer life expectancy, at least in women, and maybe also in men. Thus, the analysis of the functionality status and diversity of the rearranged TRB genes repertoire in defined T cell subsets in specific contexts will probably contribute to the understanding of the role of the immune system in defined pathophysiological conditions and aging.

## Data availability statement

Publicly available datasets were analysed in this study. This data can be found here: https://clients.adaptivebiotech.com/pub/Dean-2015-GenomeMed; TRB repertoires from ref 29 will be made available after reasonable request to the authors. The codes used for data pre-processing and analysis are available at https://github.com/ZAEDPolSl/TCR_diversity.

## Ethics statement

Ethical approval was not required for the study involving humans in accordance with the local legislation and institutional requirements. Written informed consent to participate in this study was not required from the participants or the participants’ legal guardians/next of kin in accordance with the national legislation and the institutional requirements.

## Author contributions

JM, JP: study design, repertoire modelling; all authors: results analysis. SC, JM: writing of the first draft of the manuscript; all authors: manuscript reviewing and editing. All authors contributed to the article and approved the submitted version.

## Footnotes

1
https://clients.adaptivebiotech.com/pub/Dean-2015-GenomeMed

2
https://www.imgt.org/IMGTrepertoire/LocusGenes/#F
