# Supplementary material for: Sex- and age-specific aspects of human peripheral T-cell dynamics
Source: Front Immunol. 2023 Oct 13;14:1224304. doi: 10.3389/fimmu.2023.1224304 (PMC10613070; doi:10.3389/fimmu.2023.1224304)
Supplement: Supplementary file 1 [file DataSheet_1.docx]

**Supplementary Materials**
Sex-specific aspects of human peripheral T-cell repertoire aging

Justyna Mika^1^, Kengo Yoshida^2^, Yoichiro Kusunoki^3^, Serge M. Candéias^4*^, Joanna Polanska^5*^

**Supplementary Information 1**

**Downsampling of sequences in longitudinal set regarding CD4/CD8 ratio in blood.**

The T lymphocyte CD4/CD8 ratio have been established for each donor at each time point when sorting cells (Yoshida et al., *Exp Gerontol* 2017, **96**:29-37.)

| Sample ID_time point | CD4/CD8 ratio (*BloodRatio*) |
| --- | --- |
| D1_1 | 1,004 |
| D1_2 | 0,909 |
| D1_3 | 0,830 |
| D2_1 | 2,314 |
| D2_2 | 2,277 |
| D2_3 | 3,398 |
| D3_1 | 4,054 |
| D4_1 | 2,265 |
| D4_2 | 1,945 |
| D4_3 | 1,575 |
| D5_1 | 1,586 |
| D5_2 | 1,652 |
| D5_3 | 0,724 |
| D6_1 | 1,293 |

Sequencing was performed separately for CD4(+) cells and CD8(+). In order to be able to compare cross-sectional and longitudinal sets, downsampling procedure was developed to recreate whole blood rearranged TRB repertoire for each sample using the CD4/CD8 ratio of that sample.

First the expected number of CD4 (*xCD4*) and CD8 (*xCD8*) sequences was calculated, based on the respective representation of CD4+ and CD8+ cells (*BloodRatio*)

xCD4 = BloodRatio x nCD8

xCD8 = nCD4 / BloodRatio

Where nCD4 – total number of sequences in CD4(+) sample, nCD8 – total number of sequences in CD8(+) sample.

If expected number of CD4 sequences was smaller than actually observed (*xCD4 < nCD4*), then downsampling of CD4 subset was performed in order to obtain the expected number of rearranged TRB sequences from CD4+ cells. In the opposite situation, expected number of CD8 sequences is smaller than the actually observed number of CD8 sequences (*xCD8 < nCD8*), thus in this case, downsampling of CD8 subset was performed.

The downsampling was performed with the *sample()* function in R language, by sampling without the replacement an expected number of sequences from known distribution of all sequenced rearrangements (ie. if a clone had 100 copies, than it had 100 chances to be sampled from the distribution).

**Supplementary Figure 1**

Supplementary Figure 1 shows the distribution of total counts of sequences (which is equivalent to sequencing coverage) after data curation.


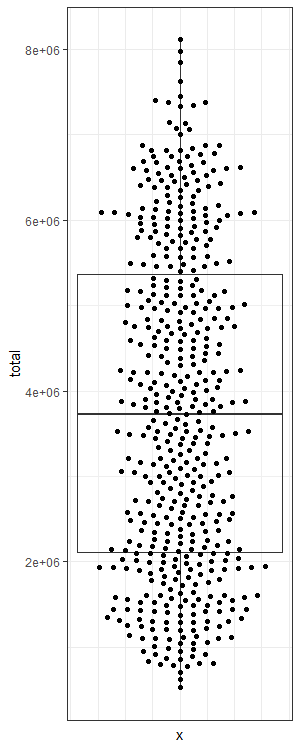


**Supplementary Table 1**

Supplementary Table 1 shows the deciles of the total counts of sequences (all, productive and nonproductive) after data curation.

| **decile** | **all** | productive | nonproductive |
| --- | --- | --- | --- |
| **0%** | **526 247** | 443 805 | 82 442 |
| **10%** | **1 381 239** | 1 177 274 | 198 248 |
| **20%** | **1 913 960** | 1 632 532 | 280 961 |
| **30%** | **2 380 678** | 1 996 769 | 354 523 |
| **40%** | **3 027 118** | 2 556 599 | 447 449 |
| **50%** | **3 725 893** | 3 126 944 | 542 532 |
| **60%** | **4 320 966** | 3 623 242 | 632 769 |
| **70%** | **4 985 280** | 4 232 309 | 749 247 |
| **80%** | **5 790 392** | 4 865 355 | 849 810 |
| **90%** | **6 418 546** | 5 442 064 | 1 010 938 |
| **100%** | **8 109 134** | 7 215 219 | 1 395 823 |

All samples have at least 500,000 of sequences. More than 90% of samples have more than 1 million of sequences, about 80% of donors have more than 2 million of sequences and around 50% of donors have more than 4 million of sequences.

**Supplementary Figure 2**

Supplementary Figure 2 A-G shows the models of Status Diversity in age created for different sequencing coverages. The models were created as described in Methods subsection – Response modelling.

For every panel, there are 6 plots provided. Plots a, b and c show the weighted linear regression model for all donors (n=487), only men (n=259) and only women (n=228), respectively. Panels d, e, and f show piecewise weighted linear regression for all donors, only men, and only women respectively. The red line on all plots shows the evolution of Status Diversity as calculated by Eq.1.

**A – coverage 10,000**
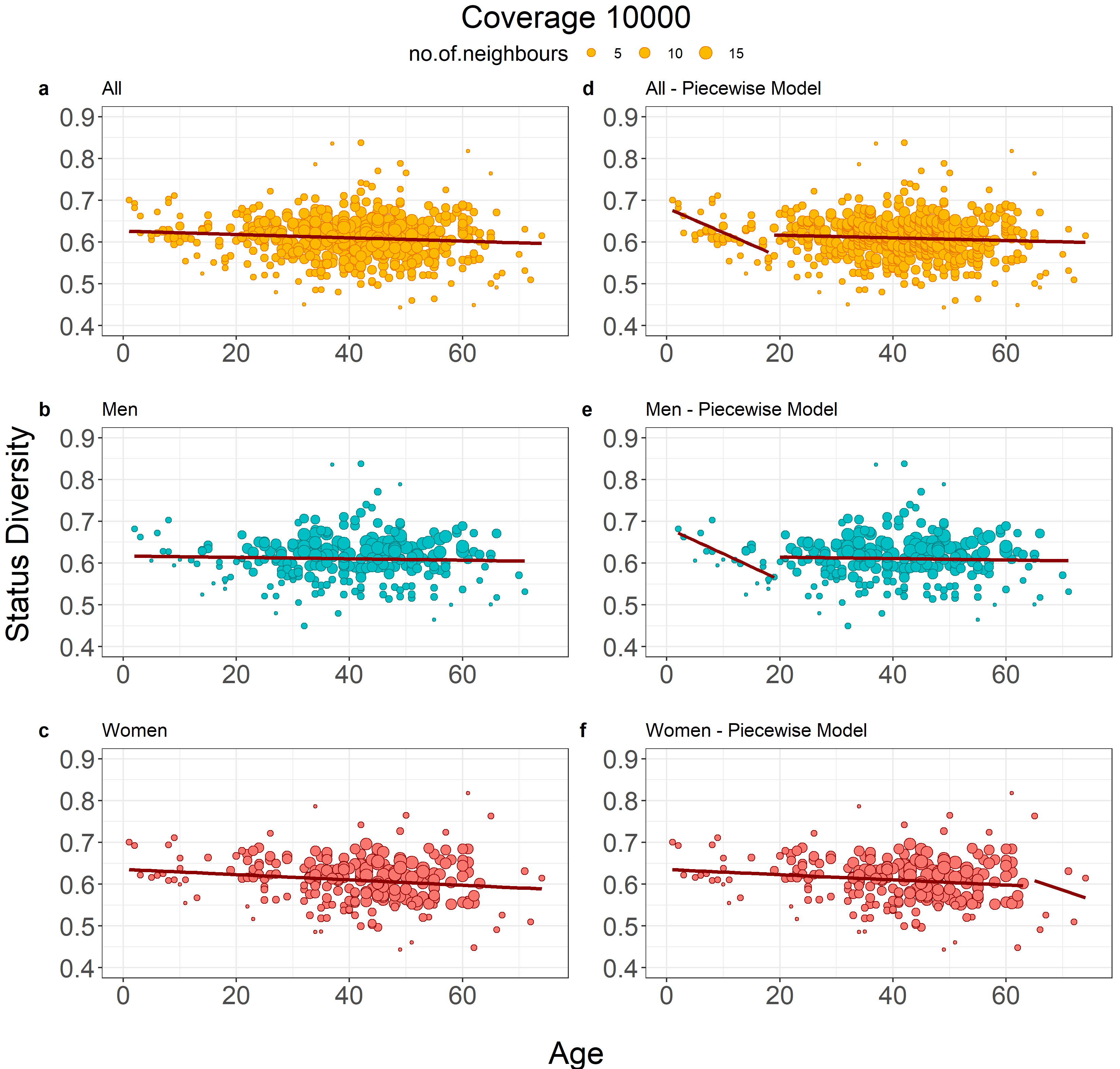


**B – coverage 80,000**


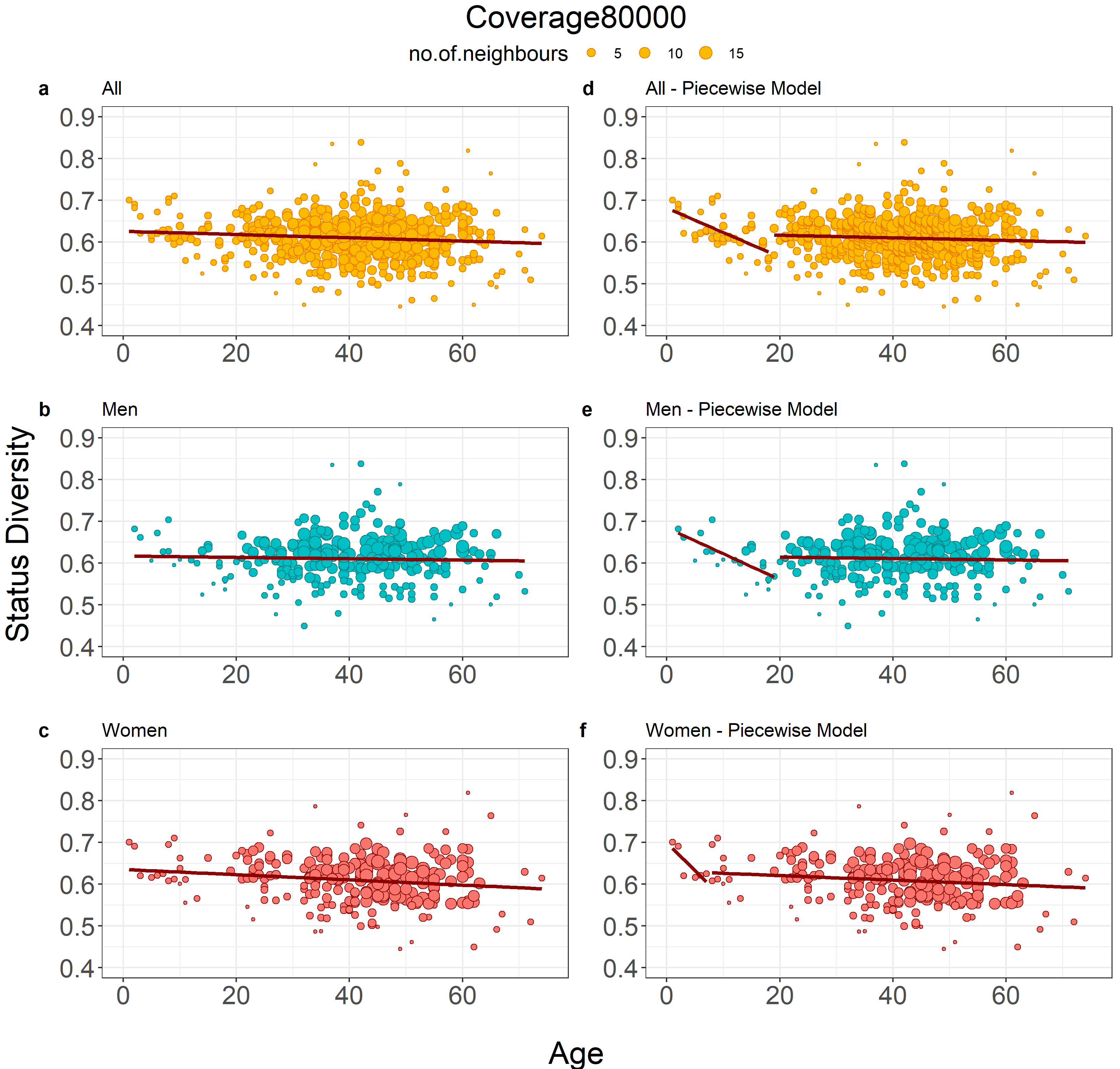


**C – coverage 150,000**


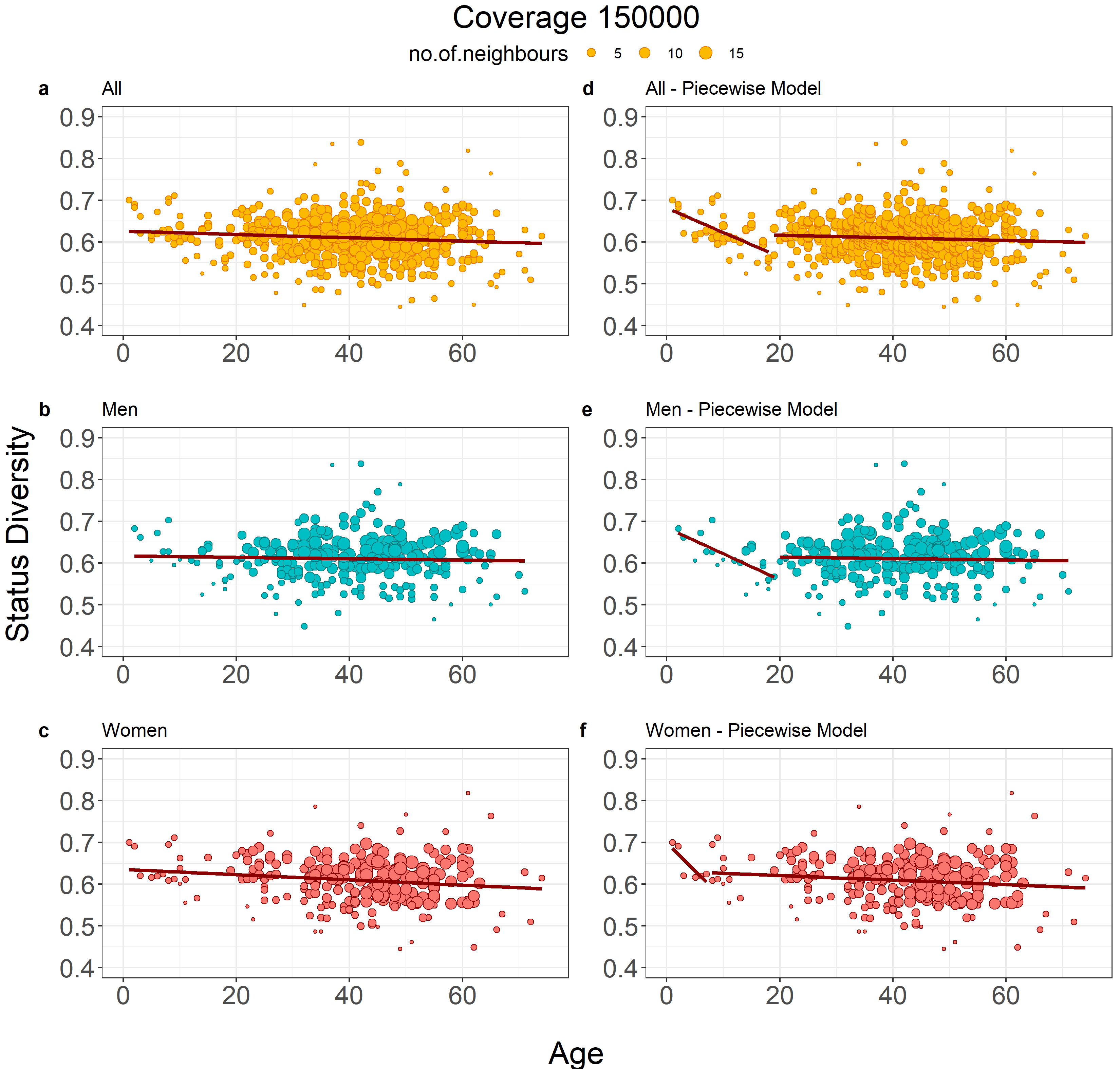


**D – coverage 500,000**


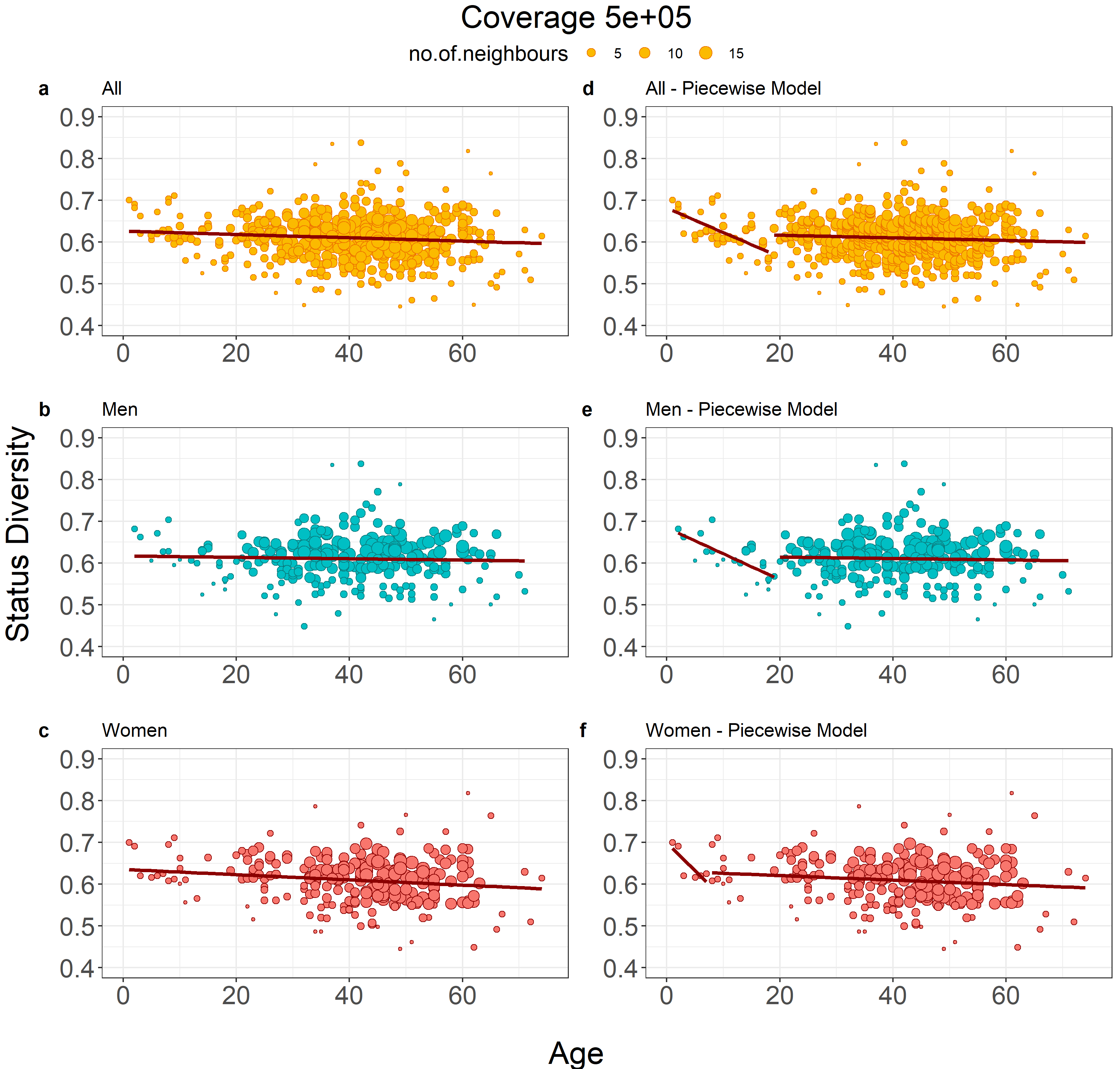


**E – coverage 1,000,000**


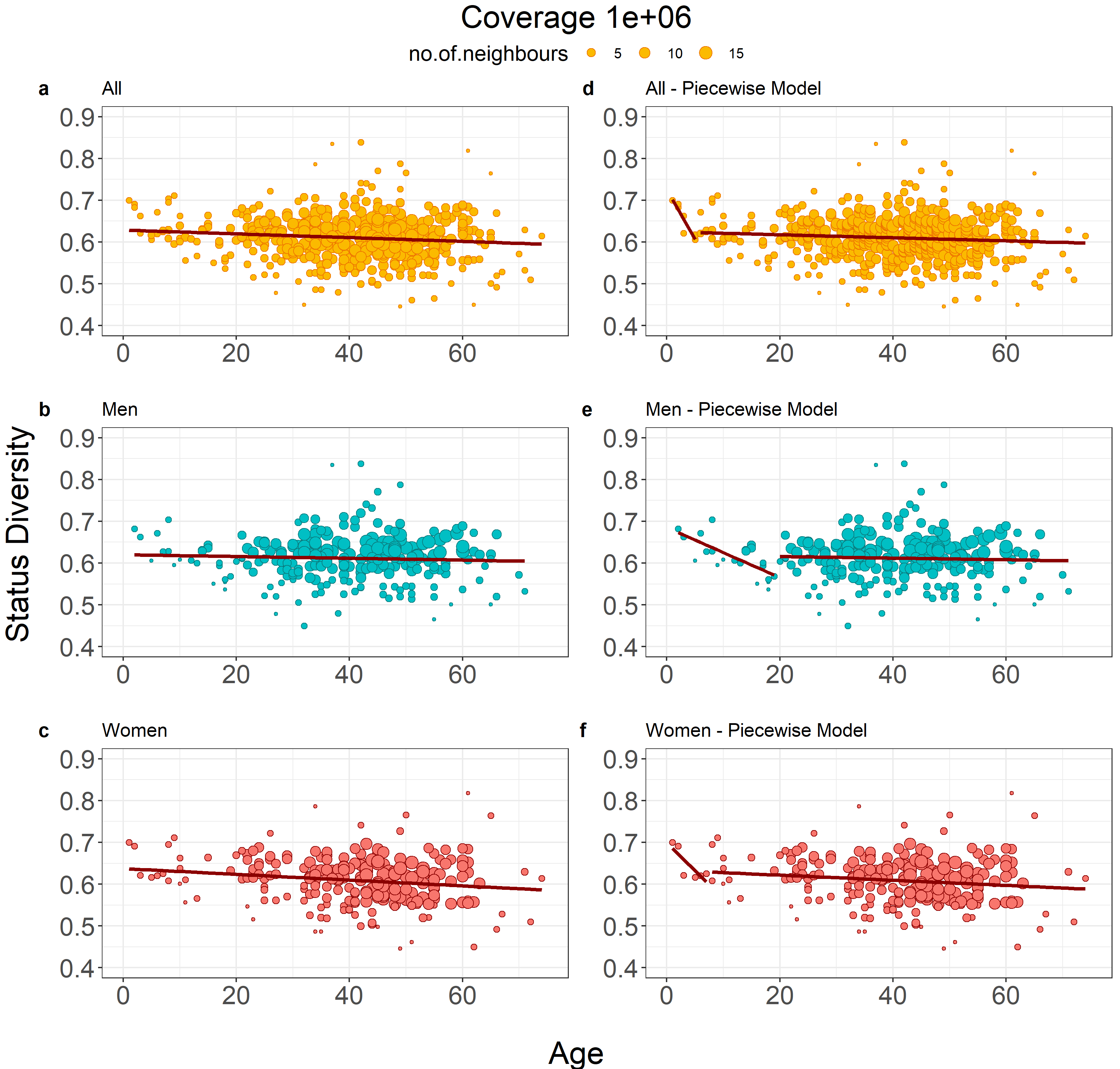


**F – coverage 2,000,000**


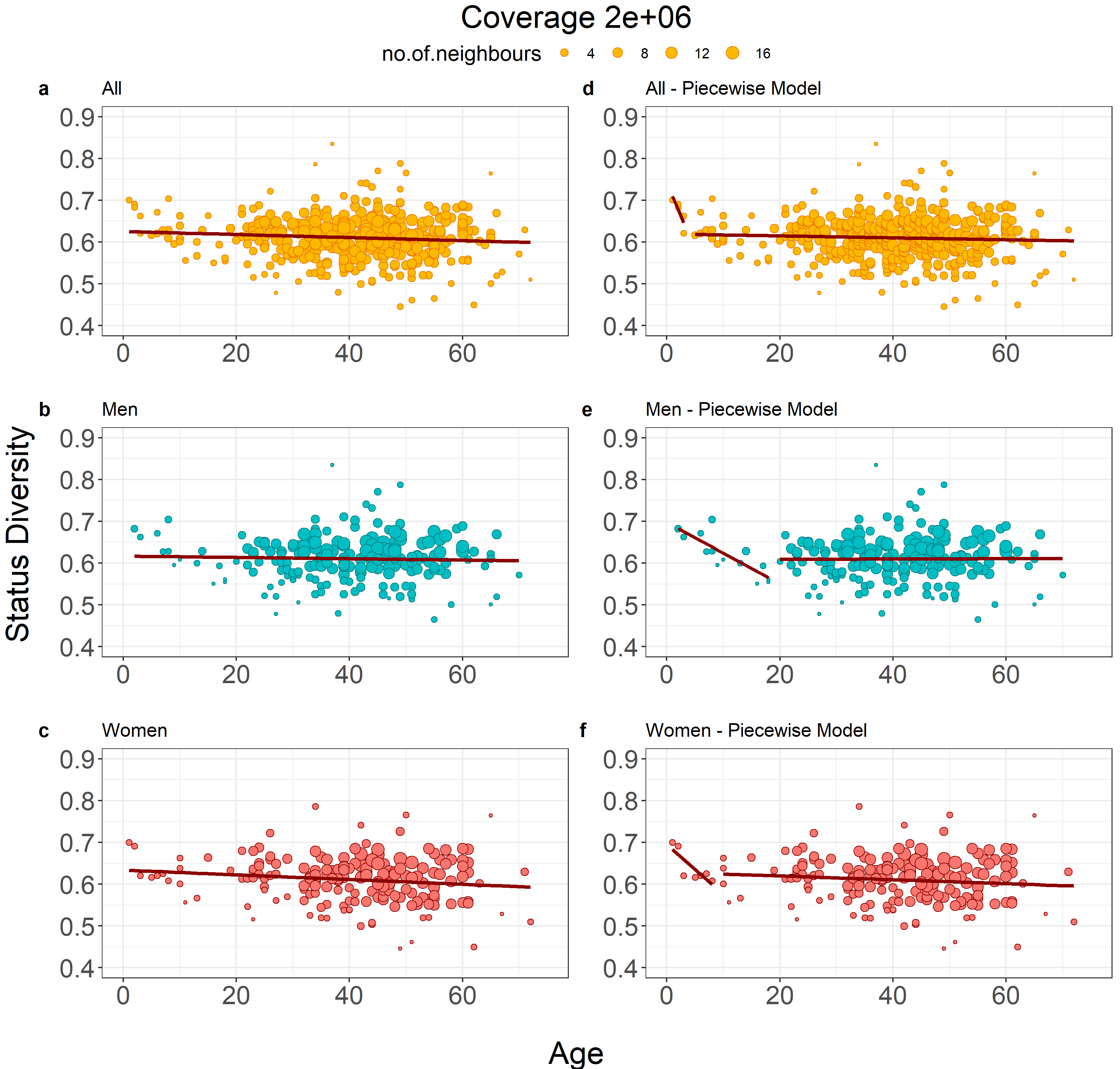


**G – coverage 4,000,000**


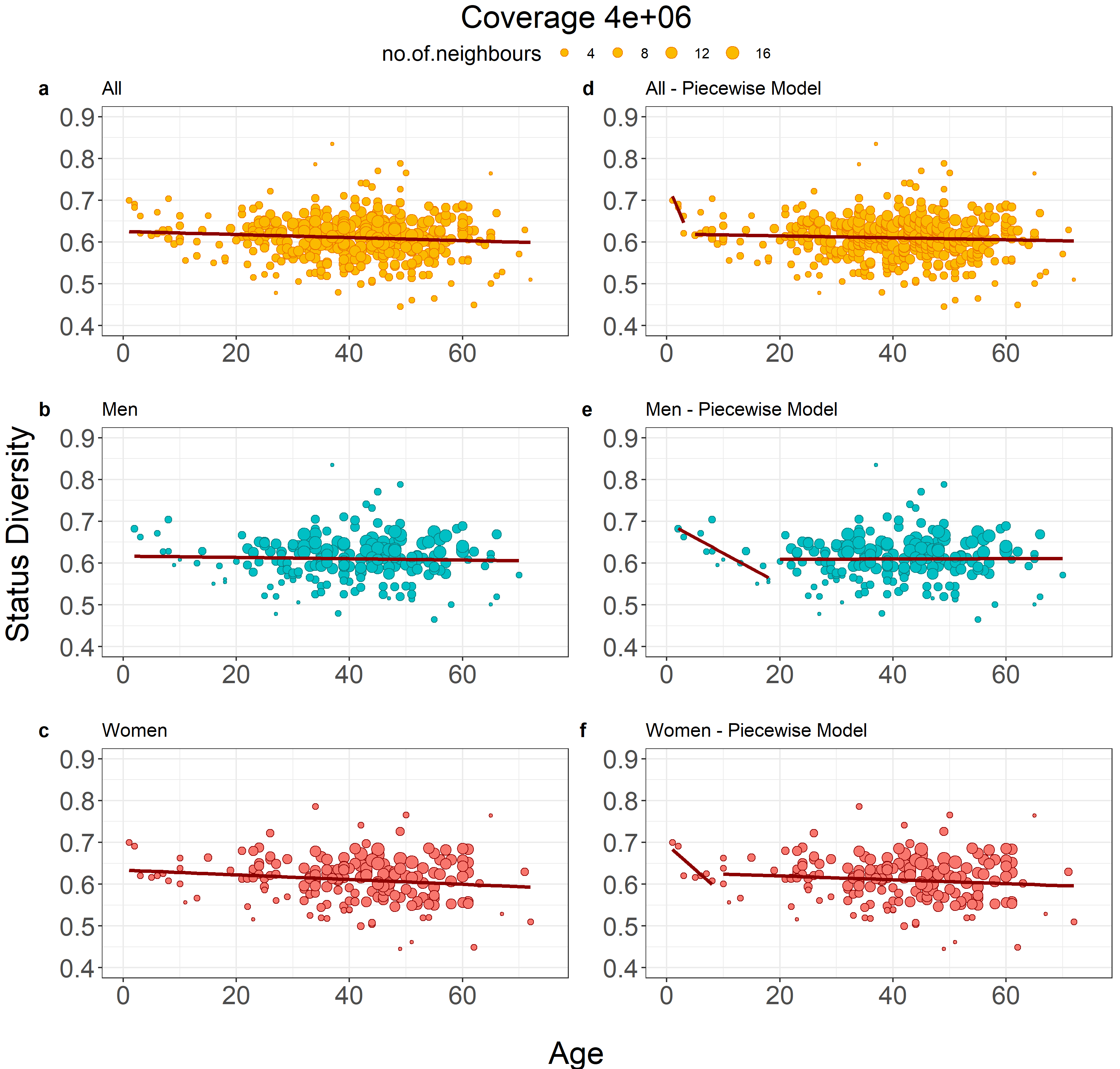


**Supplementary Table 2**

Supplementary Table 2A and B show the results of Status Diversity modelling created by linear regression model and piecewise linear regression models. The significant p-values are colored with green. 95% confidence intervals were calculated for b0 (intercept) and b1 (slope) coefficients. r corresponds to Pearson correlation coefficient and n corresponds to the number of donors for which specific model was created.

Supplementary Table 2A. Status Diversity modelling results - linear regression models

| **b0** | **95% CI - down** | **95% CI - up** | **b1** | **95% CI - down** | **95% CI - up** | **model p.value** | **r** | **n** | **coverage** | **model** |
| --- | --- | --- | --- | --- | --- | --- | --- | --- | --- | --- |
| 0.62594 | 0.61076 | 0.64112 | -0.00040 | -0.00076 | -0.00004 | 0.03006 | -0.09831 | 487 | 10000 | ONLY  AGE |
| 0.62575 | 0.61058 | 0.64092 | -0.00039 | -0.00075 | -0.00003 | 0.03287 | -0.09671 | 487 | 80000 |  |
| 0.62585 | 0.61067 | 0.64102 | -0.00039 | -0.00075 | -0.00003 | 0.03180 | -0.09731 | 487 | 150000 |  |
| 0.62587 | 0.61070 | 0.64104 | -0.00039 | -0.00075 | -0.00003 | 0.03167 | -0.09738 | 487 | 5.00E+05 |  |
| 0.62851 | 0.61305 | 0.64396 | -0.00045 | -0.00082 | -0.00008 | 0.01587 | -0.11132 | 469 | 1.00E+06 |  |
| 0.62522 | 0.60826 | 0.64218 | -0.00037 | -0.00077 | 0.00003 | 0.07332 | -0.09222 | 378 | 2.00E+06 |  |
| 0.62558 | 0.60438 | 0.64679 | -0.00028 | -0.00078 | 0.00022 | 0.27041 | -0.07480 | 219 | 4.00E+06 |  |
| 0.63559 | 0.61316 | 0.65802 | -0.00063 | -0.00115 | -0.00011 | 0.01735 | -0.15745 | 228 | 10000 | ONLY  WOMEN |
| 0.63525 | 0.61285 | 0.65765 | -0.00062 | -0.00114 | -0.00010 | 0.01883 | -0.15547 | 228 | 80000 |  |
| 0.63532 | 0.61292 | 0.65773 | -0.00063 | -0.00115 | -0.00011 | 0.01853 | -0.15585 | 228 | 150000 |  |
| 0.63535 | 0.61295 | 0.65774 | -0.00063 | -0.00115 | -0.00011 | 0.01853 | -0.15586 | 228 | 5.00E+05 |  |
| 0.63742 | 0.61465 | 0.66020 | -0.00069 | -0.00122 | -0.00016 | 0.01096 | -0.17084 | 221 | 1.00E+06 |  |
| 0.63372 | 0.60930 | 0.65813 | -0.00057 | -0.00114 | 0.00000 | 0.05161 | -0.14613 | 178 | 2.00E+06 |  |
| 0.63932 | 0.61106 | 0.66758 | -0.00053 | -0.00119 | 0.00012 | 0.10830 | -0.15762 | 105 | 4.00E+06 |  |
| 0.61679 | 0.59601 | 0.63757 | -0.00017 | -0.00067 | 0.00033 | 0.51309 | -0.04082 | 259 | 10000 | ONLY  MEN |
| 0.61674 | 0.59596 | 0.63752 | -0.00016 | -0.00066 | 0.00034 | 0.52597 | -0.03958 | 259 | 80000 |  |
| 0.61685 | 0.59606 | 0.63764 | -0.00017 | -0.00067 | 0.00034 | 0.51713 | -0.04043 | 259 | 150000 |  |
| 0.61688 | 0.59609 | 0.63766 | -0.00017 | -0.00067 | 0.00034 | 0.51539 | -0.04060 | 259 | 5.00E+05 |  |
| 0.61992 | 0.59874 | 0.64110 | -0.00021 | -0.00072 | 0.00029 | 0.40787 | -0.05279 | 248 | 1.00E+06 |  |
| 0.61662 | 0.59276 | 0.64047 | -0.00016 | -0.00073 | 0.00041 | 0.58307 | -0.03904 | 200 | 2.00E+06 |  |
| 0.61200 | 0.57997 | 0.64403 | -0.00002 | -0.00079 | 0.00076 | 0.96740 | -0.00387 | 114 | 4.00E+06 |  |

Supplementary Table 2B. Status Diversity modelling results - piecewise linear regression models

| **b0** | **95% CI - down** | **95% CI - up** | **b1** | **95% CI - down** | **95% CI - up** | **model p.value** | **r** | **n** | **coverage** | **model** |
| --- | --- | --- | --- | --- | --- | --- | --- | --- | --- | --- |
| 0.68149 | 0.65242 | 0.71057 | -0.00587 | -0.00845 | -0.00329 | 0.00005 | -0.60342 | 39 | 10000 | YOUNG ALL |
| 0.68121 | 0.65218 | 0.71024 | -0.00584 | -0.00842 | -0.00326 | 0.00005 | -0.60220 | 39 | 80000 |  |
| 0.68115 | 0.65204 | 0.71027 | -0.00583 | -0.00842 | -0.00324 | 0.00005 | -0.60029 | 39 | 150000 |  |
| 0.68117 | 0.65209 | 0.71026 | -0.00583 | -0.00842 | -0.00325 | 0.00005 | -0.60072 | 39 | 5.00E+05 |  |
| 0.72481 | 0.68584 | 0.76378 | -0.02360 | -0.03535 | -0.01185 | 0.00357 | -0.91765 | 7 | 1.00E+06 |  |
| 0.74103 | 0.65317 | 0.82889 | -0.03169 | -0.06950 | 0.00612 | 0.07586 | -0.83869 | 5 | 2.00E+06 |  |
| 0.72336 | 0.65708 | 0.78965 | -0.02356 | -0.04616 | -0.00096 | 0.04515 | -0.88643 | 5 | 4.00E+06 |  |
| 0.62255 | 0.60198 | 0.64312 | -0.00032 | -0.00079 | 0.00015 | 0.18331 | -0.06298 | 448 | 10000 | OLD ALL |
| 0.62225 | 0.60169 | 0.64281 | -0.00031 | -0.00078 | 0.00016 | 0.19603 | -0.06120 | 448 | 80000 |  |
| 0.62236 | 0.60180 | 0.64292 | -0.00031 | -0.00078 | 0.00016 | 0.19193 | -0.06176 | 448 | 150000 |  |
| 0.62239 | 0.60184 | 0.64295 | -0.00031 | -0.00078 | 0.00016 | 0.19119 | -0.06187 | 448 | 5.00E+05 |  |
| 0.62457 | 0.60809 | 0.64105 | -0.00036 | -0.00075 | 0.00002 | 0.06594 | -0.08562 | 462 | 1.00E+06 |  |
| 0.61908 | 0.60111 | 0.63705 | -0.00023 | -0.00065 | 0.00019 | 0.28500 | -0.05550 | 373 | 2.00E+06 |  |
| 0.61746 | 0.59422 | 0.64071 | -0.00010 | -0.00065 | 0.00044 | 0.71138 | -0.02544 | 214 | 4.00E+06 |  |
| 0.69996 | 0.63843 | 0.76149 | -0.01342 | -0.02695 | 0.00012 | 0.05125 | -0.80898 | 6 | 10000 | YOUNG WOMEN |
| 0.69882 | 0.63732 | 0.76032 | -0.01327 | -0.02680 | 0.00026 | 0.05282 | -0.80597 | 6 | 80000 |  |
| 0.69882 | 0.63753 | 0.76011 | -0.01334 | -0.02682 | 0.00014 | 0.05153 | -0.80844 | 6 | 150000 |  |
| 0.69859 | 0.63702 | 0.76015 | -0.01325 | -0.02679 | 0.00030 | 0.05322 | -0.80522 | 6 | 5.00E+05 |  |
| 0.69858 | 0.63716 | 0.76000 | -0.01324 | -0.02675 | 0.00027 | 0.05292 | -0.80578 | 6 | 1.00E+06 |  |
| 0.69867 | 0.63710 | 0.76025 | -0.01327 | -0.02681 | 0.00028 | 0.05299 | -0.80564 | 6 | 2.00E+06 |  |
| 0.67801 | 0.60728 | 0.74874 | -0.00670 | -0.01980 | 0.00640 | 0.22859 | -0.57896 | 6 | 4.00E+06 |  |
| 0.63174 | 0.60657 | 0.65692 | -0.00055 | -0.00113 | 0.00003 | 0.06177 | -0.12558 | 222 | 10000 | OLD WOMEN |
| 0.63147 | 0.60632 | 0.65661 | -0.00054 | -0.00112 | 0.00003 | 0.06509 | -0.12402 | 222 | 80000 |  |
| 0.63164 | 0.60649 | 0.65678 | -0.00055 | -0.00112 | 0.00003 | 0.06337 | -0.12482 | 222 | 150000 |  |
| 0.63163 | 0.60649 | 0.65677 | -0.00055 | -0.00112 | 0.00003 | 0.06363 | -0.12470 | 222 | 5.00E+05 |  |
| 0.63404 | 0.60836 | 0.65973 | -0.00062 | -0.00121 | -0.00003 | 0.04072 | -0.13969 | 215 | 1.00E+06 |  |
| 0.62821 | 0.59985 | 0.65657 | -0.00045 | -0.00110 | 0.00021 | 0.17860 | -0.10304 | 172 | 2.00E+06 |  |
| 0.63160 | 0.59560 | 0.66761 | -0.00037 | -0.00118 | 0.00044 | 0.36873 | -0.09131 | 99 | 4.00E+06 |  |
| 0.68483 | 0.64625 | 0.72341 | -0.00624 | -0.00919 | -0.00330 | 0.00024 | -0.69342 | 23 | 10000 | YOUNG MEN |
| 0.68454 | 0.64591 | 0.72316 | -0.00621 | -0.00916 | -0.00326 | 0.00026 | -0.69097 | 23 | 80000 |  |
| 0.68471 | 0.64595 | 0.72347 | -0.00622 | -0.00918 | -0.00326 | 0.00027 | -0.69044 | 23 | 150000 |  |
| 0.68470 | 0.64603 | 0.72337 | -0.00622 | -0.00917 | -0.00327 | 0.00026 | -0.69102 | 23 | 5.00E+05 |  |
| 0.68503 | 0.65002 | 0.72005 | -0.00597 | -0.00865 | -0.00329 | 0.00016 | -0.72050 | 22 | 1.00E+06 |  |
| 0.69770 | 0.66075 | 0.73466 | -0.00739 | -0.01051 | -0.00427 | 0.00024 | -0.83031 | 14 | 2.00E+06 |  |
| 0.70842 | 0.65318 | 0.76365 | -0.00833 | -0.01328 | -0.00337 | 0.00470 | -0.80783 | 10 | 4.00E+06 |  |
| 0.61747 | 0.58921 | 0.64573 | -0.00017 | -0.00082 | 0.00048 | 0.60808 | -0.03355 | 236 | 10000 | OLD MEN |
| 0.61732 | 0.58905 | 0.64559 | -0.00016 | -0.00082 | 0.00049 | 0.62317 | -0.03215 | 236 | 80000 |  |
| 0.61747 | 0.58920 | 0.64574 | -0.00017 | -0.00082 | 0.00049 | 0.61421 | -0.03298 | 236 | 150000 |  |
| 0.61748 | 0.58921 | 0.64575 | -0.00017 | -0.00082 | 0.00049 | 0.61351 | -0.03304 | 236 | 5.00E+05 |  |
| 0.61923 | 0.59030 | 0.64816 | -0.00019 | -0.00085 | 0.00048 | 0.57778 | -0.03722 | 226 | 1.00E+06 |  |
| 0.60803 | 0.57682 | 0.63925 | 0.00004 | -0.00068 | 0.00076 | 0.91532 | 0.00785 | 186 | 2.00E+06 |  |
| 0.59210 | 0.54778 | 0.63642 | 0.00043 | -0.00059 | 0.00145 | 0.40559 | 0.08241 | 104 | 4.00E+06 |  |

Coverage values have very small impact on the average Status Diversity and its models in time.

Coverage for up to 1 million sequences, confirms the findings, which were observed for original data. For higher coverage, 2 million and 4 million sequences, where respectively 22% and 55% of samples were discarded, only models for young men and young women are significant.

**Supplementary Figure 3**

Supplementary Figure 3 A-G shows the models of Sequence Diversity in age created for different sequencing coverages. The models were created as described in Methods subsection – Response modelling.

For every panel, there are 6 plots provided. Plots a, b and c show the weighted linear regression model for all donors (n=487), only men (n=259) and only women (n=228), respectively. Panels d, e, and f show piecewise weighted linear regression for all donors, only men, and only women respectively. The red line on all plots shows the evolution of Sequence Diversity as calculated by Eq.2 and Eq.3.

**A – coverage 10,000**


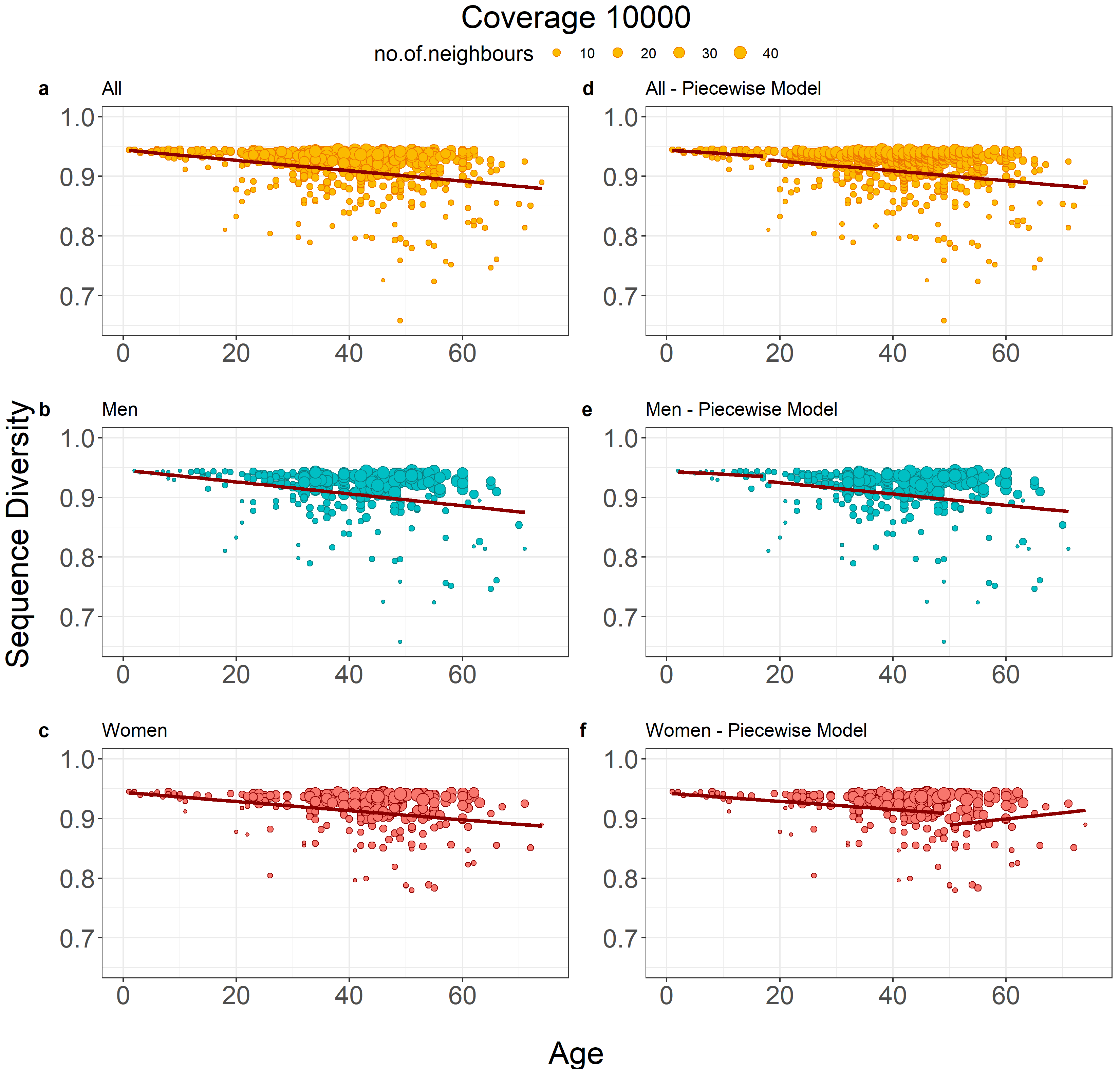


**B – coverage 80,000**


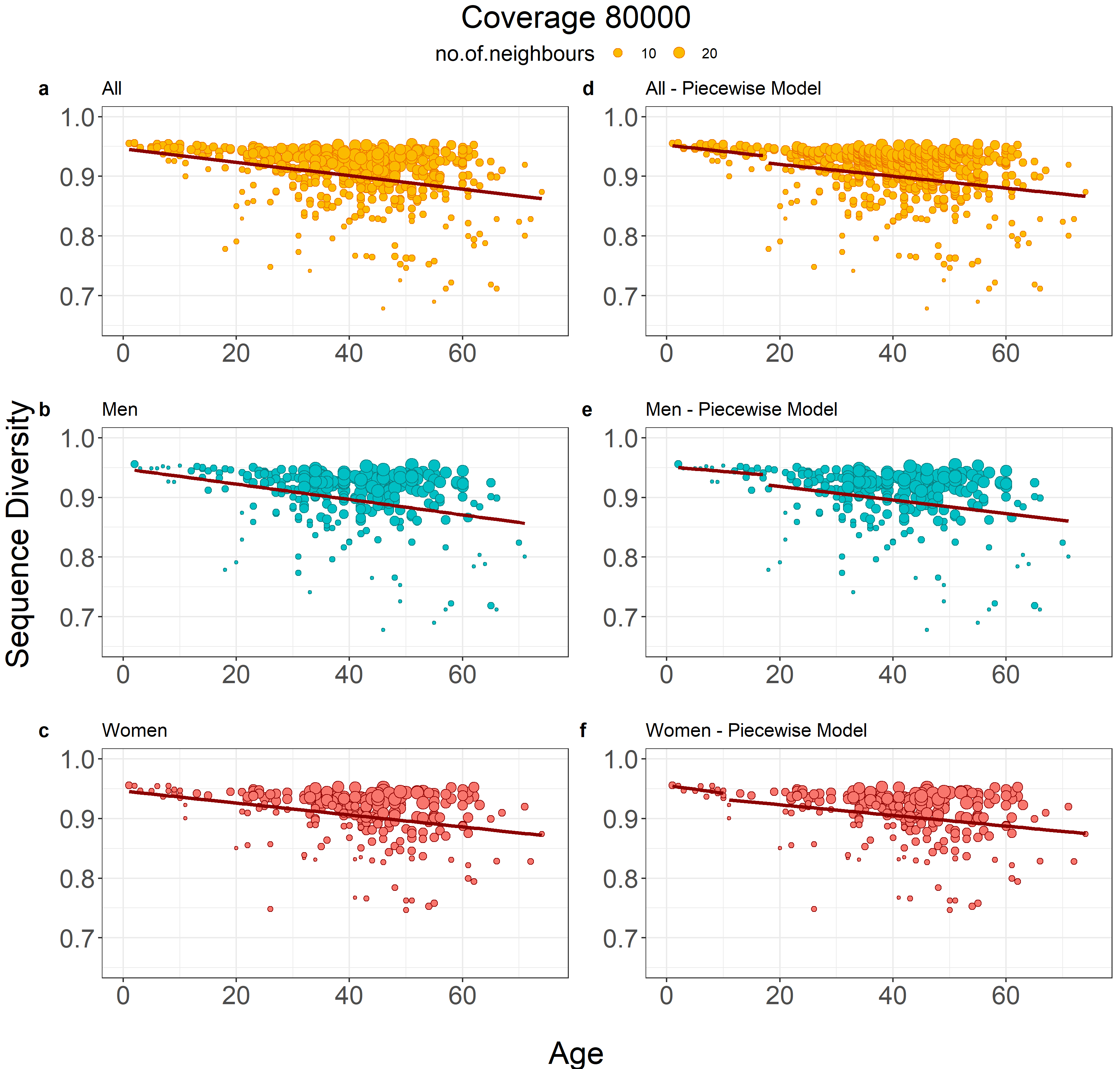


**C – coverage 150,000**


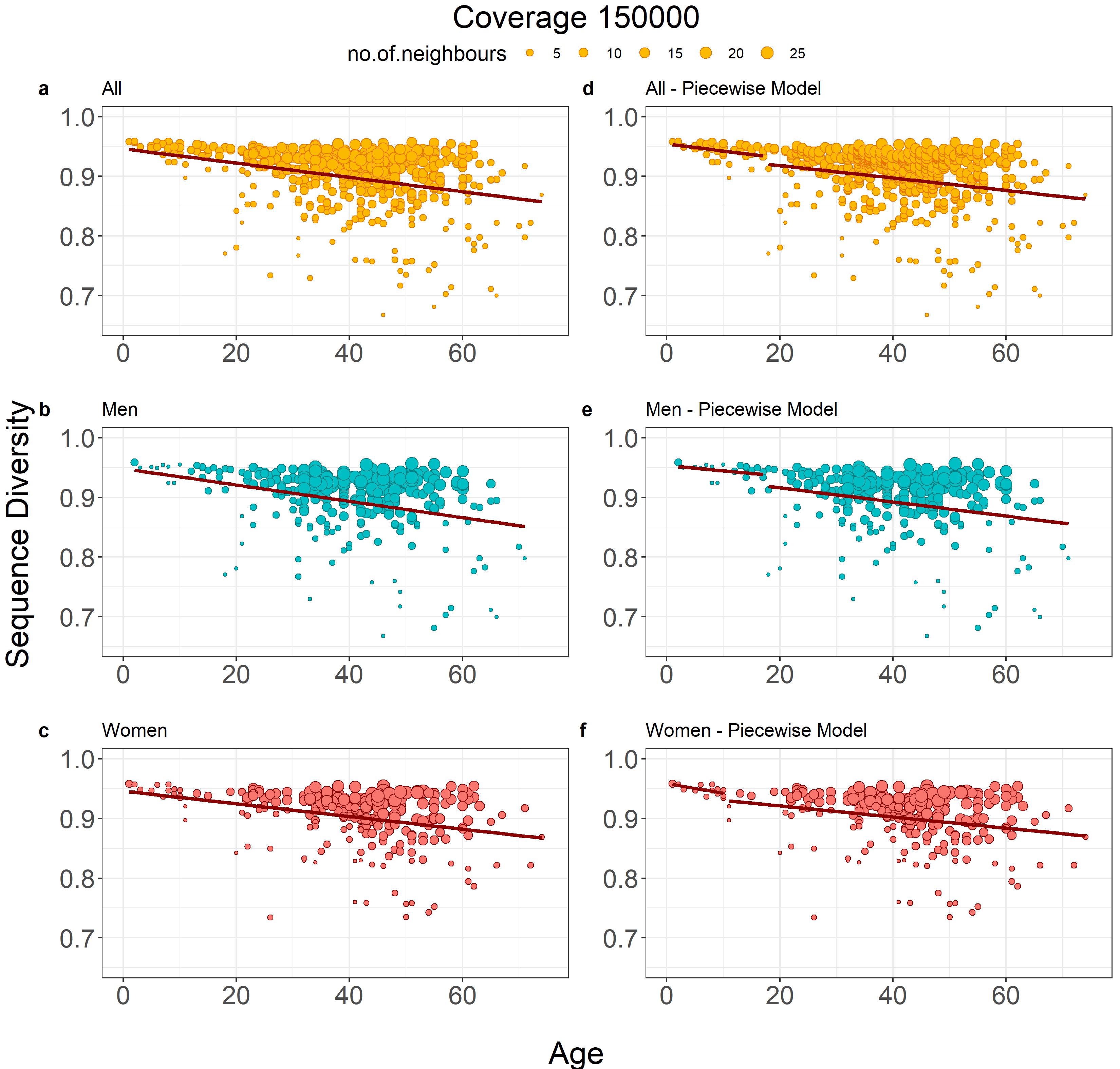


**D – coverage 500,000**


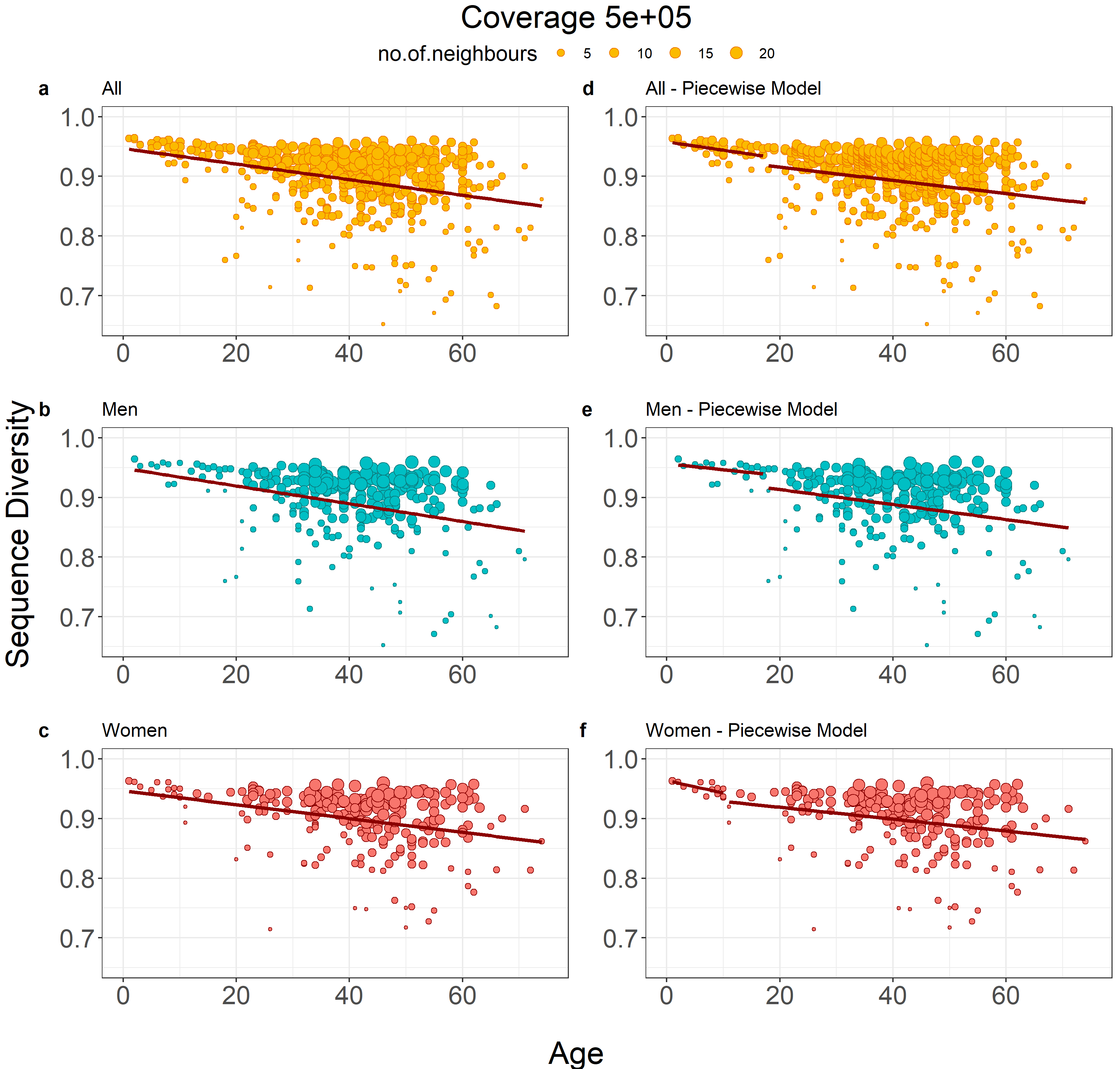


**E – coverage 1,000,000**


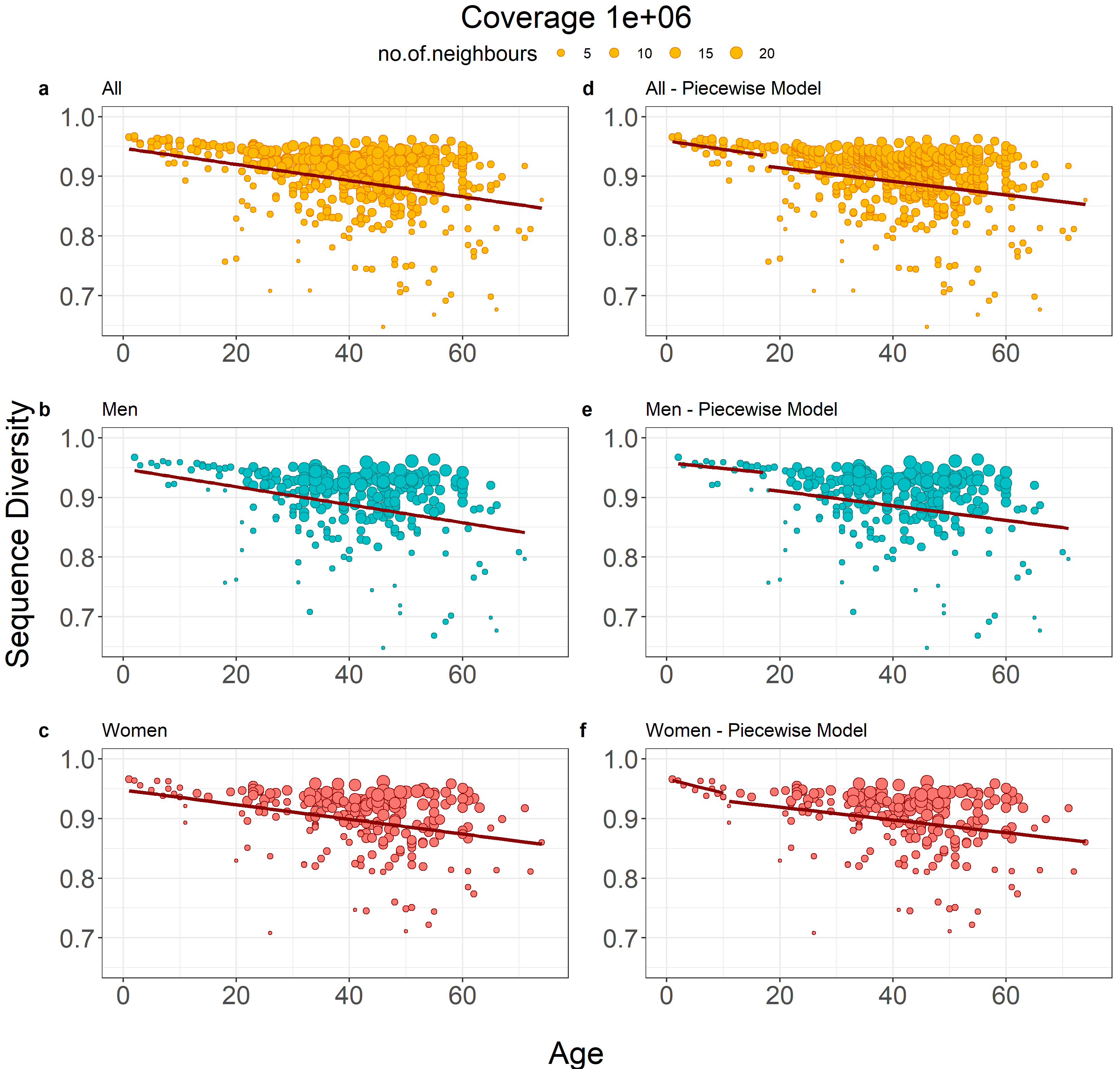


**F – coverage 2,000,000**


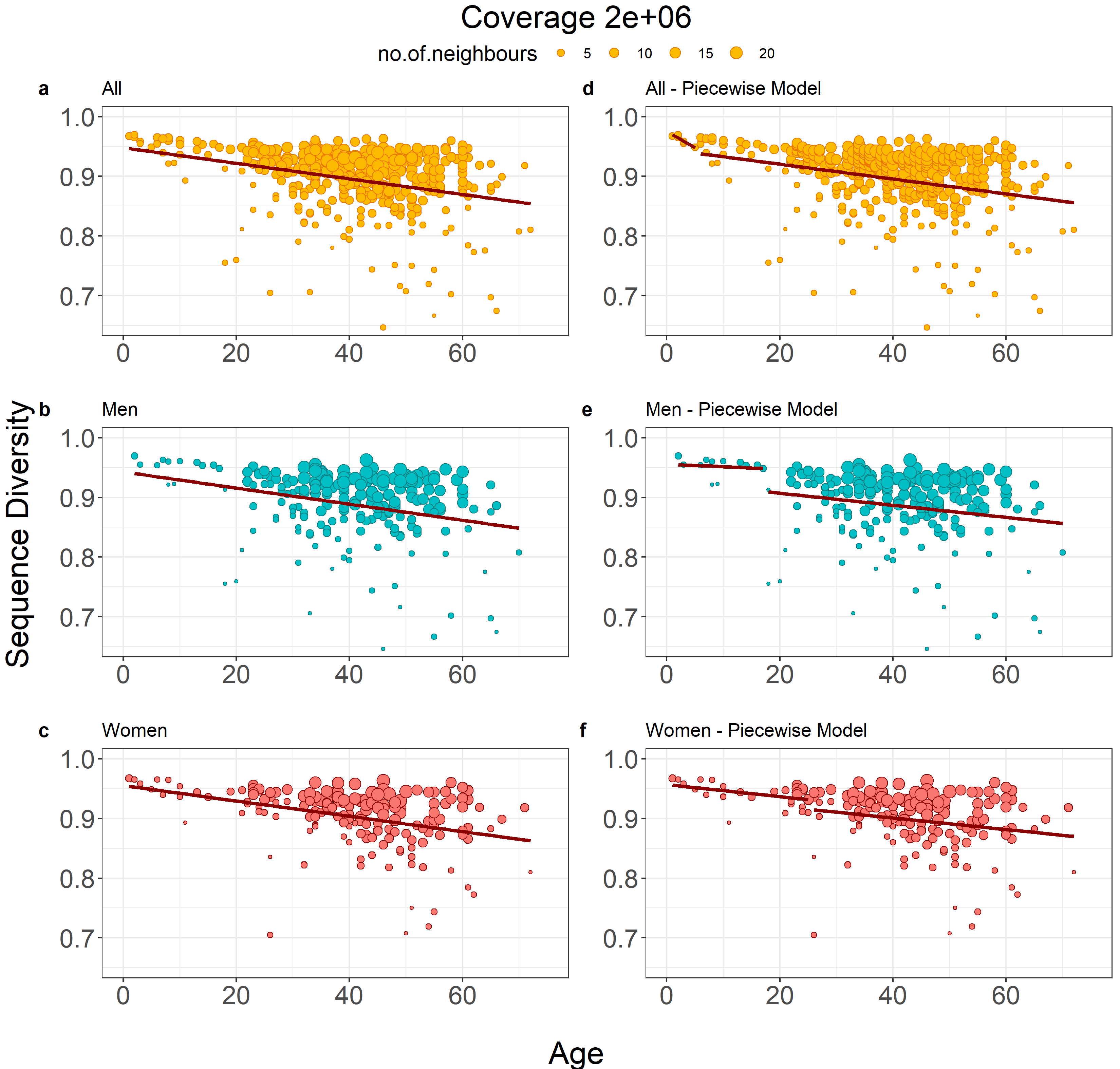


**G – coverage 4,000,000**


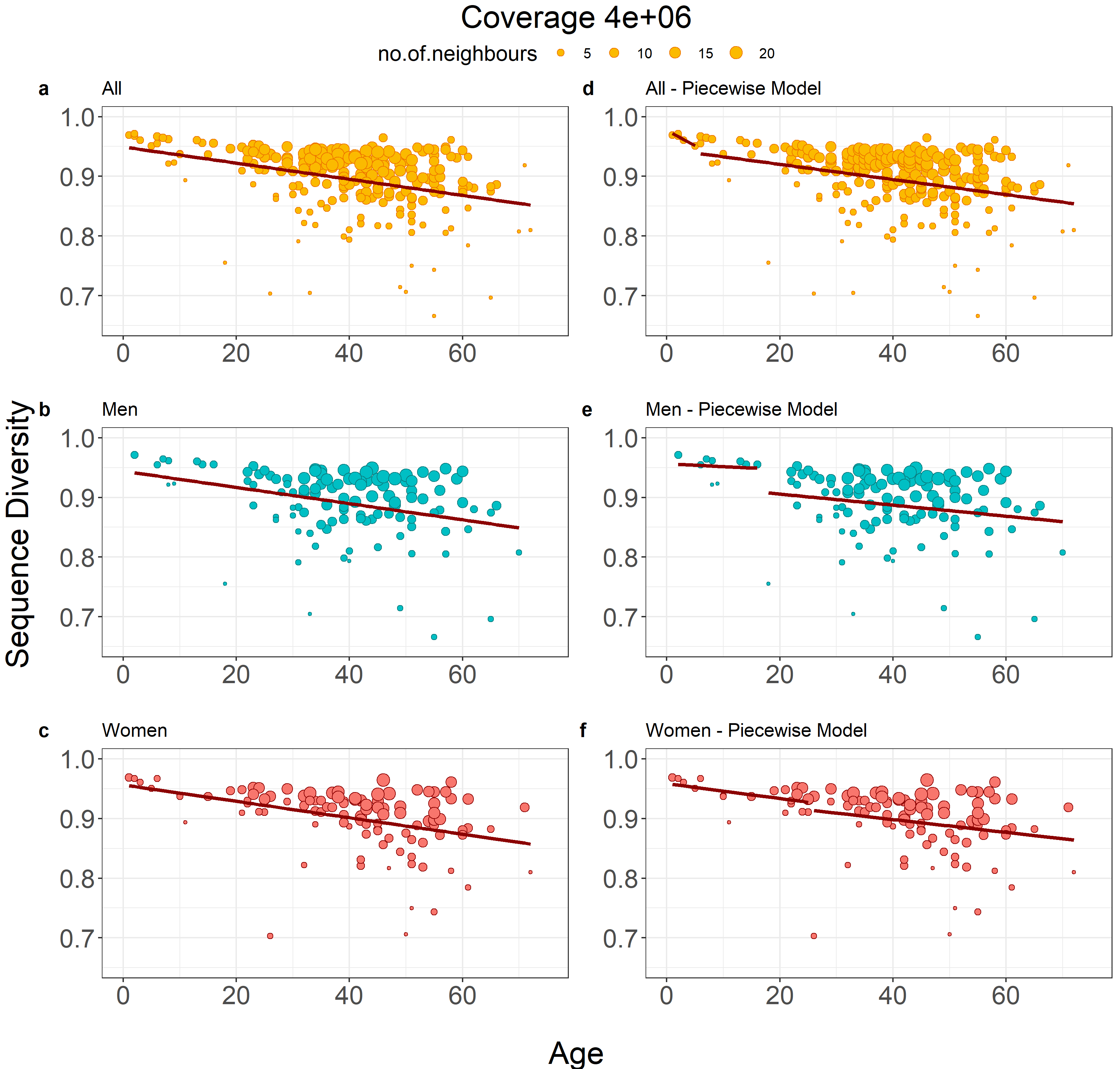


**Supplementary Table 3**

Supplementary Table 3A and B show the results of SEQUENCE Diversity modelling created by linear regression model and piecewise linear regression models. The significant p-values are colored with green. 95% confidence intervals were calculated for b0 (intercept) and b1 (slope) coefficients. r corresponds to Pearson correlation coefficient and n corresponds to the number of donors for which specific model was created.

Supplementary Table 3A. Sequence Diversity modelling results - linear regression models

| **b0** | **95% CI - down** | **95% CI - up** | **b1** | **95% CI - down** | **95% CI - up** | **model p.value** | **r** | **n** | **coverage** | **model** |
| --- | --- | --- | --- | --- | --- | --- | --- | --- | --- | --- |
| 0.9444 | 0.9338 | 0.9549 | -0.0009 | -0.0011 | -0.0006 | 2.14E-11 | -0.2973 | 487 | 10000 | ONLY  AGE |
| 0.9463 | 0.9333 | 0.9593 | -0.0011 | -0.0014 | -0.0008 | 2.10E-12 | -0.3113 | 487 | 80000 |  |
| 0.9463 | 0.9327 | 0.9598 | -0.0012 | -0.0015 | -0.0009 | 8.79E-13 | -0.3164 | 487 | 150000 |  |
| 0.9468 | 0.9325 | 0.9611 | -0.0013 | -0.0016 | -0.0010 | 2.04E-13 | -0.3247 | 487 | 5.00E+05 |  |
| 0.9472 | 0.9322 | 0.9622 | -0.0014 | -0.0017 | -0.0010 | 3.10E-13 | -0.3281 | 469 | 1.00E+06 |  |
| 0.9480 | 0.9318 | 0.9641 | -0.0013 | -0.0017 | -0.0009 | 7.78E-11 | -0.3264 | 378 | 2.00E+06 |  |
| 0.9493 | 0.9293 | 0.9694 | -0.0014 | -0.0018 | -0.0009 | 5.68E-08 | -0.3567 | 219 | 4.00E+06 |  |
| 0.9439 | 0.9307 | 0.9571 | -0.0008 | -0.0011 | -0.0005 | 1.63E-06 | -0.3113 | 228 | 10000 | ONLY  WOMEN |
| 0.9460 | 0.9294 | 0.9627 | -0.0010 | -0.0014 | -0.0006 | 7.54E-07 | -0.3207 | 228 | 80000 |  |
| 0.9460 | 0.9285 | 0.9635 | -0.0011 | -0.0015 | -0.0007 | 5.45E-07 | -0.3245 | 228 | 150000 |  |
| 0.9464 | 0.9278 | 0.9651 | -0.0012 | -0.0016 | -0.0007 | 3.09E-07 | -0.3311 | 228 | 5.00E+05 |  |
| 0.9479 | 0.9284 | 0.9673 | -0.0012 | -0.0017 | -0.0008 | 2.21E-07 | -0.3400 | 221 | 1.00E+06 |  |
| 0.9551 | 0.9351 | 0.9752 | -0.0013 | -0.0018 | -0.0008 | 2.00E-07 | -0.3778 | 178 | 2.00E+06 |  |
| 0.9564 | 0.9300 | 0.9828 | -0.0014 | -0.0020 | -0.0008 | 2.07E-05 | -0.4026 | 105 | 4.00E+06 |  |
| 0.9460 | 0.9299 | 0.9622 | -0.0010 | -0.0014 | -0.0006 | 8.05E-07 | -0.3009 | 259 | 10000 | ONLY  MEN |
| 0.9482 | 0.9286 | 0.9678 | -0.0013 | -0.0018 | -0.0008 | 1.75E-07 | -0.3177 | 259 | 80000 |  |
| 0.9483 | 0.9279 | 0.9687 | -0.0014 | -0.0019 | -0.0009 | 1.00E-07 | -0.3236 | 259 | 150000 |  |
| 0.9490 | 0.9275 | 0.9704 | -0.0015 | -0.0020 | -0.0010 | 4.01E-08 | -0.3330 | 259 | 5.00E+05 |  |
| 0.9480 | 0.9255 | 0.9705 | -0.0015 | -0.0020 | -0.0010 | 9.93E-08 | -0.3305 | 248 | 1.00E+06 |  |
| 0.9428 | 0.9178 | 0.9677 | -0.0013 | -0.0019 | -0.0008 | 1.41E-05 | -0.3017 | 200 | 2.00E+06 |  |
| 0.9440 | 0.9136 | 0.9744 | -0.0014 | -0.0021 | -0.0006 | 3.69E-04 | -0.3279 | 114 | 4.00E+06 |  |

Supplementary Table 3B. Sequence Diversity modelling results - piecewise linear regression models

| **b0** | **95% CI - down** | **95% CI - up** | **b1** | **95% CI - down** | **95% CI - up** | **model p.value** | **r** | **n** | **coverage** | **model** |
| --- | --- | --- | --- | --- | --- | --- | --- | --- | --- | --- |
| 0.9438 | 0.9379 | 0.9498 | -0.0006 | -0.0011 | 0.0000 | 4.43E-02 | -0.3372 | 36 | 10000 | YOUNG ALL |
| 0.9524 | 0.9430 | 0.9619 | -0.0010 | -0.0019 | -0.0001 | 2.40E-02 | -0.3756 | 36 | 80000 |  |
| 0.9543 | 0.9439 | 0.9647 | -0.0012 | -0.0022 | -0.0002 | 2.12E-02 | -0.3827 | 36 | 150000 |  |
| 0.9578 | 0.9460 | 0.9696 | -0.0014 | -0.0025 | -0.0003 | 1.71E-02 | -0.3949 | 36 | 5.00E+05 |  |
| 0.9595 | 0.9468 | 0.9722 | -0.0014 | -0.0026 | -0.0002 | 2.39E-02 | -0.3867 | 34 | 1.00E+06 |  |
| 0.9751 | 0.9656 | 0.9846 | -0.0054 | -0.0087 | -0.0022 | 9.39E-03 | -0.9198 | 6 | 2.00E+06 |  |
| 0.9770 | 0.9671 | 0.9869 | -0.0052 | -0.0085 | -0.0018 | 1.66E-02 | -0.9421 | 5 | 4.00E+06 |  |
| 0.9425 | 0.9281 | 0.9568 | -0.0008 | -0.0012 | -0.0005 | 8.88E-07 | -0.2290 | 451 | 10000 | OLD ALL |
| 0.9402 | 0.9225 | 0.9578 | -0.0010 | -0.0014 | -0.0006 | 1.62E-06 | -0.2236 | 451 | 80000 |  |
| 0.9389 | 0.9205 | 0.9573 | -0.0010 | -0.0015 | -0.0006 | 1.59E-06 | -0.2238 | 451 | 150000 |  |
| 0.9375 | 0.9181 | 0.9570 | -0.0011 | -0.0016 | -0.0007 | 1.37E-06 | -0.2251 | 451 | 5.00E+05 |  |
| 0.9370 | 0.9168 | 0.9572 | -0.0011 | -0.0016 | -0.0007 | 1.87E-06 | -0.2263 | 435 | 1.00E+06 |  |
| 0.9454 | 0.9280 | 0.9629 | -0.0012 | -0.0017 | -0.0008 | 5.19E-09 | -0.2970 | 372 | 2.00E+06 |  |
| 0.9455 | 0.9233 | 0.9677 | -0.0013 | -0.0018 | -0.0007 | 2.69E-06 | -0.3145 | 214 | 4.00E+06 |  |
| 0.9424 | 0.9292 | 0.9556 | -0.0007 | -0.0010 | -0.0003 | 2.79E-04 | -0.2777 | 167 | 10000 | YOUNG WOMEN |
| 0.9556 | 0.9468 | 0.9644 | -0.0013 | -0.0025 | -0.0001 | 3.37E-02 | -0.5901 | 13 | 80000 |  |
| 0.9586 | 0.9487 | 0.9685 | -0.0016 | -0.0029 | -0.0003 | 2.28E-02 | -0.6234 | 13 | 150000 |  |
| 0.9638 | 0.9520 | 0.9755 | -0.0021 | -0.0037 | -0.0005 | 1.45E-02 | -0.6580 | 13 | 5.00E+05 |  |
| 0.9666 | 0.9535 | 0.9797 | -0.0024 | -0.0042 | -0.0006 | 1.44E-02 | -0.6827 | 12 | 1.00E+06 |  |
| 0.9568 | 0.9427 | 0.9710 | -0.0010 | -0.0018 | -0.0002 | 1.24E-02 | -0.4507 | 30 | 2.00E+06 |  |
| 0.9586 | 0.9412 | 0.9760 | -0.0013 | -0.0022 | -0.0003 | 1.34E-02 | -0.5305 | 21 | 4.00E+06 |  |
| 0.8377 | 0.7159 | 0.9594 | 0.0010 | -0.0011 | 0.0032 | 3.37E-01 | 0.1251 | 61 | 10000 | OLD WOMEN |
| 0.9410 | 0.9194 | 0.9625 | -0.0009 | -0.0014 | -0.0004 | 3.79E-04 | -0.2402 | 215 | 80000 |  |
| 0.9399 | 0.9173 | 0.9624 | -0.0009 | -0.0014 | -0.0004 | 3.93E-04 | -0.2396 | 215 | 150000 |  |
| 0.9388 | 0.9148 | 0.9629 | -0.0010 | -0.0015 | -0.0005 | 3.74E-04 | -0.2404 | 215 | 5.00E+05 |  |
| 0.9406 | 0.9158 | 0.9654 | -0.0011 | -0.0016 | -0.0005 | 2.19E-04 | -0.2530 | 209 | 1.00E+06 |  |
| 0.9395 | 0.9025 | 0.9766 | -0.0010 | -0.0018 | -0.0002 | 1.87E-02 | -0.1932 | 148 | 2.00E+06 |  |
| 0.9414 | 0.8854 | 0.9975 | -0.0011 | -0.0023 | 0.0001 | 7.38E-02 | -0.1961 | 84 | 4.00E+06 |  |
| 0.9440 | 0.9350 | 0.9530 | -0.0005 | -0.0013 | 0.0003 | 1.78E-01 | -0.3225 | 19 | 10000 | YOUNG MEN |
| 0.9517 | 0.9379 | 0.9656 | -0.0008 | -0.0020 | 0.0004 | 1.70E-01 | -0.3285 | 19 | 80000 |  |
| 0.9533 | 0.9381 | 0.9685 | -0.0009 | -0.0022 | 0.0004 | 1.73E-01 | -0.3259 | 19 | 150000 |  |
| 0.9566 | 0.9394 | 0.9738 | -0.0010 | -0.0025 | 0.0005 | 1.69E-01 | -0.3292 | 19 | 5.00E+05 |  |
| 0.9583 | 0.9401 | 0.9765 | -0.0010 | -0.0025 | 0.0006 | 2.11E-01 | -0.3096 | 18 | 1.00E+06 |  |
| 0.9559 | 0.9331 | 0.9787 | -0.0004 | -0.0026 | 0.0017 | 6.62E-01 | -0.1410 | 12 | 2.00E+06 |  |
| 0.9564 | 0.9202 | 0.9926 | -0.0005 | -0.0040 | 0.0031 | 7.67E-01 | -0.1155 | 9 | 4.00E+06 |  |
| 0.9436 | 0.9221 | 0.9651 | -0.0009 | -0.0014 | -0.0004 | 2.36E-04 | -0.2352 | 240 | 10000 | OLD MEN |
| 0.9412 | 0.9151 | 0.9672 | -0.0011 | -0.0017 | -0.0005 | 2.77E-04 | -0.2327 | 240 | 80000 |  |
| 0.9399 | 0.9128 | 0.9669 | -0.0012 | -0.0018 | -0.0006 | 2.61E-04 | -0.2337 | 240 | 150000 |  |
| 0.9384 | 0.9100 | 0.9669 | -0.0013 | -0.0019 | -0.0006 | 2.28E-04 | -0.2358 | 240 | 5.00E+05 |  |
| 0.9351 | 0.9054 | 0.9648 | -0.0012 | -0.0019 | -0.0005 | 5.45E-04 | -0.2263 | 230 | 1.00E+06 |  |
| 0.9274 | 0.8952 | 0.9595 | -0.0010 | -0.0018 | -0.0003 | 8.25E-03 | -0.1922 | 188 | 2.00E+06 |  |
| 0.9242 | 0.8824 | 0.9660 | -0.0009 | -0.0019 | 0.00004 | 6.14E-02 | -0.1832 | 105 | 4.00E+06 |  |

The lower the coverage, the higher on average the Sequence Diversity.

There is no big impact of the coverage on the Sequence Diversity models and their significance.

With higher coverage, there were more samples discarded (due to the small coverage in original samples). For coverage of 2 million and 4 million sequences, the piecewise regression model for women resulted in different age split, than the remaining one (however here 22% and 55% of samples were discarded).
